# N-terminal lipidation enhances membrane interaction and antifungal activity of a Trematocine-derived decapeptide

**DOI:** 10.1007/s00253-026-13929-4

**Published:** 2026-06-29

**Authors:** Damiano Squitieri, Federica Massaro, Melinda Mariotti, Margherita Cacaci, Stefano Borocci, Fernando Porcelli, Francesco Buonocore, Francesca Bugli, Maurizio Sanguinetti

**Affiliations:** 1 Department of Basic Biotechnological Sciences, Intensive and Perioperative Clinics, Catholic University of the Sacred Heart, L.Go Francesco Vito 1, 00168 Rome, Italy; 2 Department for Innovation in Biological, Agro-Food and Forest Systems (DIBAF), University of Tuscia, L.Go Dell’Università S.N.C, 01100 Viterbo, Italy; 3 Department of Laboratory Sciences and Infectious Diseases, A, Gemelli University Hospital Foundation IRCCS, L.Go Agostino Gemelli 8, 00168 Rome, Italy

**Keywords:** Lipidated antimicrobial peptides, Antifungal resistance, Antibiofilm activity, Yeasts

## Abstract

**Abstract:**

Invasive fungal infections are a growing global health threat, driven by the limited availability of antifungal therapies and the rapid spread of multidrug-resistant yeasts. This study explored the effects of N-terminal lipidation on Trem-4, a decapeptide derived from the antimicrobial peptide Trematocine-HSK, to enhance its antifungal potential. Trem-4 was modified with caprylic (C8) and myristic (C14) fatty acid chains, generating two lipopeptides: Cap-T4 and Myr-T4. Biophysical analyses, including fluorescence-based assays and circular dichroism, showed that lipidation significantly improved peptide–membrane interactions compared to the non-lipidated form. The two derivatives displayed distinct behaviors, with differences in aggregation and membrane selectivity depending on acyl chain length. Antifungal activity was assessed against 60 clinically characterized yeast isolates, including *Candida* spp., *Candidozyma haemuli complex*, and *Cryptococcus neoformans*, under both planktonic and biofilm conditions. Both lipopeptides exhibited broad-spectrum activity, against clinically relevant species, with conserved activity against antifungal-resistant strains. Importantly, both compounds remained effective against mature biofilms, causing metabolic disruption and structural damage. Cap-T4 demonstrated consistent activity across species, while Myr-T4 showed enhanced potency against selected isolates, particularly *C. auris*. Biocompatibility evaluation revealed a concentration-dependent cytotoxicity and hemolysis, more pronounced for Myr-T4. However, both compounds were well tolerated in vivo in the *Galleria mellonella* model. Overall, Cap-T4 emerged as the most promising candidate for further preclinical development. These findings support N-terminal lipidation as an effective strategy to improve the antifungal efficacy of short antimicrobial peptides and highlight its potential for developing new treatments against drug-resistant fungal pathogens.

**Graphical abstract:**

**Figure:**
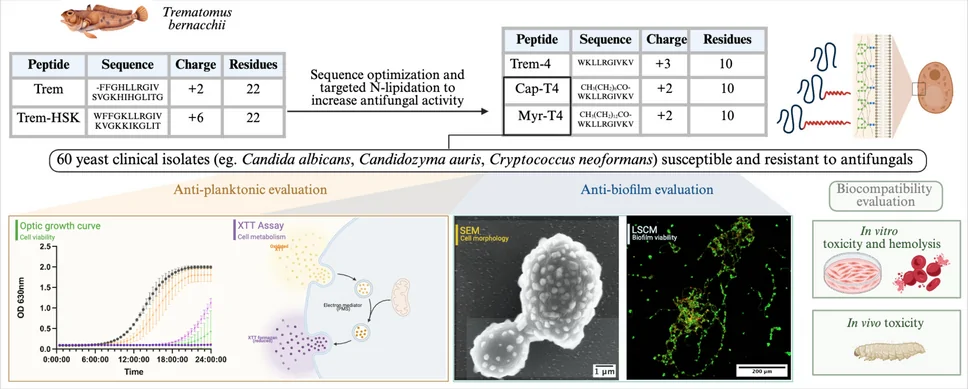

**Key points:**

- *N-terminal lipidation enhances Trem-4 membrane interaction and activity.*
- *Myr-T4 and Cap-T4 show broad activity against susceptible and resistant yeasts.*
- *Cap-T4 retains activity against yeasts and mature biofilms.*

**Supplementary Information:**

The online version contains supplementary material available at https://doi.org/10.1007/s00253-026-13929-4.

## Introduction

Invasive fungal infections (IFIs), defined as deep-seated and systemic infections caused predominantly by opportunistic yeasts and molds, have increased significantly in recent decades and represent a growing public health concern (Webb et al. 2018). IFIs are associated with high morbidity and mortality, particularly among critically ill patients admitted to intensive care units, and occur most frequently in individuals affected by cancer, AIDS or subjected to long-term immunosuppressive therapies (Trelles et al. 2025).

Yeasts of the genus *Candida* represent the most frequently reported causative agents of IFIs worldwide, followed by infections caused by *Cryptococcus* species (Fang et al. 2023).

This scenario is further aggravated by the increasing prevalence of strains resistant to antifungal agents (Denning 2024). Many fungal species have developed resistance to all four main classes of antifungal drugs: azoles, polyenes, pyrimidine analogs, and echinocandins (Revie et al. 2018).

As a consequence of this phenomenon, the annual number of deaths due to fungal infections has increased significantly, from 1.6 million per year globally in 2017 to 3.75 million in 2023, with 2.55 million directly attributable to fungal disease (Bongomin et al. 2017; Denning 2024).

To address this emergency, the World Health Organization in 2022 published a list of priority fungal pathogens, aiming to guide research, development and public health interventions in response to antifungal resistance (World Health Organization 2022; Casalini et al. 2024). The yeasts included in the list belong to *Candida*, *Candidozyma* (formerly known as *Candida*) and *Cryptococcus* species.

Due to the structural and functional similarity between fungi and mammalian cells (for example, the organization of DNA into chromosomes within a nucleus, the presence of cytoplasmic organelles, and several conserved biosynthetic pathways), the number of antifungal drugs that can be used systemically is reduced, and is particularly challenging to develop antifungal agents with selective toxicity, as identifying molecular targets that are sufficiently distinct from those of human cells (Rautenbach et al. 2016; Fernández de Ullivarri et al. 2020). To date, therefore, the treatment of IFIs represents a major clinical challenge and makes it essential to investigate new molecules to use as an alternative or in combination with existing conventional therapy (Pappas et al. 2018). In this context, antimicrobial lipopeptides (LPs) are a particularly promising class of molecules that can be considered.

Lipopeptides, an emerging class of antimicrobial peptides (AMPs), are amphiphilic molecules composed of one or more lipid moieties conjugated to a short peptide headgroup, typically comprising up to ten amino acids (Hamley 2015; Singh and Kumar 2025).

The key process for the antifungal action of most LPs is interaction with the fungal membrane (Makovitzki et al. 2006). Particularly, the initial driving force for the association with the fungal membrane is represented by the electrostatic interaction between the cationic residues of the molecule and the negatively charged phosphate groups of the microbial membrane. This is followed by the establishment of van der Waals interactions between the acyl chains of the lipopeptide and the hydrophobic core of the membrane, with consequent partitioning of the lipopeptide inside the membrane (Li et al. 2017; Wiman et al. 2023). It has been demonstrated that lipid chains can self-associate into clusters that insert into lipid bilayers, inducing membrane curvature stress, and ultimately increasing membrane permeability (Lin et al. 2023). The lipid moieties also act as anchors, enhancing and stabilizing electrostatic and hydrophobic interactions between the peptide fraction and the cell membrane (Bugli et al. 2022).

This work explored the effect of the length of the acyl tail on the chemical-physical properties, biological and antifungal activity of a short peptide, named Trem-4 (WKLLRGIVKV), derived from the AMP Trematocine-HSK (Squitieri et al. 2024).

Specifically, Trem-4 peptide was subjected to N-terminal lipidation by the addition of caprylic and myristic fatty acid tails, of 8 and 14 carbon atoms, respectively. These two modifications provide an initial comparison between shorter and longer hydrophobic chains, and they do not fully define the relationship among acyl-chain length, lipophilicity, and antifungal activity.

The two resulting lipopeptides, named Cap-T4 and Myr-T4, were designed to possess characteristics that confer membrane-active properties: the hydrophobic portion, provided by the lipid tail and a tryptophan residue in position 1, promotes partitioning inside the hydrophobic core of the lipid bilayer, while polar amino acids with positively charged side chains facilitate electrostatic interactions with negatively charged lipids (Juhaniewicz-Dębińska et al. 2020). The N-terminus was selected as the initial lipidation site because it provides a chemically defined single-point modification without alteration of side-chain residues, particularly cationic residues that may contribute to electrostatic membrane recognition. This approach allowed comparison of lipidated analogs without changing the internal peptide sequence. Moreover, lipidation reduced the net peptide charge from + 3 to + 2, which may affect the electrostatic attraction between the peptide and negatively charged fungal membrane components. However, the biological effects result from a balance between enhanced hydrophobic membrane insertion and altered electrostatic interactions.

Also, introducing an acyl chain can substitute hydrophobic amino acids in the peptide sequence, allowing a reduction in peptide amino acid chain length and consequently enhancing in vivo stability and at the same time reducing the costs associated with their synthesis (Lin and Grossfield 2014; Oliveras et al. 2018).

Specifically, the acyl chain lengths, 8-carbon fatty acid (C8) and 14-carbon fatty acid (C14), were selected because it was observed that C8 represents a good chain length for improving the biological activity of short peptides, whereas it is reported that lipidation with C14 fatty acid promotes the appearance of antifungal activity (Húmpola et al. 2017; Rounds and Straus 2020; Bugli et al. 2022).

Longer chains, such as C16 and C18, tend to induce higher hemolytic and cytotoxic effects, while shorter acyl chains fail to provide sufficient hydrophobic contribution (Rounds and Straus 2020).

The three peptides, Trem-4, Cap-T4 and Myr-T4 (Table 1) were initially characterized by determining their critical aggregation concentration (CAC) and their interaction and affinity for anionic and zwitterionic phospholipid vesicles (LUVs) designed to mimic the cell membranes of microorganisms and mammals, respectively. Their structural characteristics were then evaluated using circular dichroism (CD) spectroscopy in solution and in the presence of the two membrane models.

**Table 1:** Physico-chemical properties of Trem-4, and the two corresponding lipidated peptides

Peptide	Sequence	Net charge	Aminoacidic residue	Molecular weight (Da)
Trem-4 Cap-T4 Myr-T4	WKLLRGIVKV CH_3_(CH_2_)_6_CO-WKLLRGIVKV CH_3_(CH_2_)_12_CO-WKLLRGIVKV	+ 3 + 2 + 2	10 10 10	1212 1356 1440

Antimicrobial efficacy was next evaluated against a broad spectrum (60 isolates) of characterized fungal clinical isolates in planktonic and biofilm forms. The efficacy was assessed on 7 different yeasts species: *Candida albicans*, *Candidozyma auris*, *Cryptococcus neoformans*, *Candida parapsilosis*, *Candida glabrata*, *Candida tropicalis* and *Candidozyma vulturna*. The antifungal characterization included phenotypic, metabolic, and microscopy-based investigations. In vitro toxicity studies against mammalian erythrocytes, murine immortalized cell line 3T3, and human primary cell line FB789 were carried out to assess their biocompatibility and possible pharmacological applications. Finally, in vivo toxicity was evaluated with the *Galleria mellonella* larvae model. To date, only a limited number of studies have evaluated novel lipopeptides against large panels of clinical fungal isolates (Ramachandran et al. 2018). In this context, our work provides a distinct contribution by investigating a rationally designed decapeptide derived from a previously described AMP originating from the innate immune system of an Arctic fish (Squitieri et al. 2024).

## Materials and methods 

### Chemicals and peptides

Chemical products were acquired from Sigma-Aldrich. POPC (1-palmitoyl-2-oleoyl-sn-glycero-3-phosphocholine) and POPG (1-palmitoyl-2-oleoyl-sn-glycero-3-phospho-1’-rac-glycerol) were bought from Avanti Polar Lipids (Alabaster, AL, USA).

Trem-4, Myr-T4, and Cap-T4 were purchased from CASLO ApS with a purity > 98% (Supplementary materials SM2–4). Concentration of the different used peptides was assigned by absorption spectroscopy (ε_280_ = 5500 M^−1^ cm^−1^).

### Lipid vesicles preparation

Large unilamellar vesicles (LUVs) formed by 100% POPC (w/w), to mimic the zwitterionic membrane, and 70%/30% (w/w) POPC-POPG, to mimic the anionic membrane, were employed. To make these systems, specific amounts of lipids were dissolved in chloroform. This solvent was removed by rotary evaporation and the samples were maintained overnight under high vacuum. The lipid film was successively hydrated with 1 mL of buffer solution (0.01 M phosphate buffer, 0.0027 M potassium chloride, 0.137 M sodium chloride, and 0.1 mM EDTA, pH 7.4) to obtain 10 mM concentration and subjected to 5 freeze-thaw cycles. The lipid suspensions were finally extruded as described before (Olivieri et al. 2018; Della Pelle et al. 2020; Squitieri et al. 2024).

### Partition constant determination

Trem-4, Cap-T4, and Myr-T4 peptides partitioning between a polar and apolar environment was studied monitoring the increase of tryptophan fluorescence inside the sequence of all peptides after increasing LUVs concentration. The range of LUVs tested concentration was different for each peptide considering the ability of peptides to interact with the membranes. Specifically, the different ranges were between 0 and 2.4 mM for Trem-4, 0 and 1.6 mM for Cap-T4, and 0 and 0.8 mM for Myr-T4.

1.0 μM concentration of each peptide was titrated with different LUVs (POPC 100% and 70%−30% POPC-POPG) and the fluorescence emissions were recorded between 315 and 400 nm with λ_exc_ = 290 nm. A scan speed of 200 nm/min was used with a bandwidth for excitation and emission of 5 and 10 nm, respectively and a cross-oriented configuration of the polarizers (*pol*_*em*_ = 0° and *pol*_*exc*_ = 90°) to reduce scattering from vesicles. Subtraction of the background buffer emission was performed for each spectrum.

Mole fraction partition coefficients (*K*_x_) were calculated from the fraction of the partitioned peptide (*f*_p_) according to the Wimley equation (Wimley 2010) (1):

1 $${f}_{p}=\frac{{\mathrm{K}}_{\mathrm{x}} [\mathrm{L}]}{\mathrm{W}+ {\mathrm{K}}_{\mathrm{x}} [\mathrm{L}]}$$

where [L] is the molar concentration of added lipid and [W] is the concentration of water, 55.5 M.

Specifically, ${\mathrm{K}}_{\mathrm{x}}$ values were determined by experimental data plotting the value of F/F_0_, as a function of the lipid concentration relative to each addition and interpolating the data using the relation:

2 $$\frac{F}{{F}_{0}}=1+(\mathrm{F}\mathrm{m}\mathrm{a}\mathrm{x}-1)\mathrm{f}\mathrm{p}$$

where F is the fluorescence intensity measured at each lipid addition and F_0_ is the fluorescence intensity of the peptide before the addition of vesicles.

From the K_x_ values the free energy of partition was calculated using the relationship (3):

3 $$\Delta \mathrm{G}^\circ =-\mathrm{R}\mathrm{T}\mathrm{l}\mathrm{n} {\mathrm{K}}_{\mathrm{x}}$$

where R is the universal gas constant, 8.314 J/(mol·K), and T is the temperature, 298.15 K.

Finally, the selectivity ratio value, defined as the ratio between the value of the partition constant measured in the presence of POPC-POPG and the value measured in the presence of 100% POPC, was calculated (4).

4 $$\mathrm{S}\mathrm{e}\mathrm{l}\mathrm{e}\mathrm{c}\mathrm{t}\mathrm{i}\mathrm{v}\mathrm{i}\mathrm{t}\mathrm{y} \mathrm{r}\mathrm{a}\mathrm{t}\mathrm{i}\mathrm{o}= \frac{{\mathrm{K}}_{\mathrm{x} } (\mathrm{P}\mathrm{O}\mathrm{P}\mathrm{C})}{{\mathrm{K}}_{\mathrm{x} } (\mathrm{P}\mathrm{O}\mathrm{P}\mathrm{C}/\mathrm{P}\mathrm{O}\mathrm{P}\mathrm{G})}$$

### Acrylamide quenching experiments

The fluorescence quenching of all peptides at a concentration of 3 µM was determined both in buffer solution (0.01 M phosphate buffer, 0.0027 M potassium chloride, and 0.137 M sodium chloride and 0.1 mM EDTA, pH 7.4) and in the presence of liposomes, by measuring the variation of tryptophan emission fluorescence upon the addition of acrylamide in a range between 0 and 255 mM.

Measurements in the presence of liposomes were performed at the end of the partition experiment, under conditions favoring peptide partitioning. Fluorescence spectra were recorded using an excitation wavelength of 290 nm and a scan speed of 200 nm/min.

The data were fitted using the Stern–Volmer equation to determine the Stern–Volmer constant (K_sv_) and Net Accessibility Factor (NAF) as described before (Squitieri et al. 2024).

### Critical aggregation concentration (CAC) determination

The CAC value of the N-terminal lipidated peptides Cap-T4 and Myr-T4 was determined in water by fluorescence spectroscopy using 8-anilinonaphthalene-1-sulfonate (ANS) as the fluorescent probe (Bugli et al. 2022).

Since the length of the acyclic chain influences the propensity of the lipopeptide to aggregate, the assays were performed using peptide concentrations from 0 to 30 μM for Myr-T4, and from 0 to 240 μM for Cap-T4, in 0.5 μM ANS solution.

The ANS was excited at a wavelength of 360 nm, and each fluorescence spectrum was recorded between 400 and 600 nm.

### Circular dichroism

Circular Dichroism (CD) spectroscopy experiments were performed using a J715 JASCO spectropolarimeter, to study the secondary structures of the three peptides in both a buffer solution (phosphate buffer 10 mM, pH 7.4 and 0.1 mM EDTA) and in the presence of the liposomes 100% POPC and 70–30% POPC-POPG as described previously (Squitieri et al. 2024).

### Fungal clinical strains

A total of 60 clinical fungal strains were characterized and tested against enquired LPs. The isolates were firstly identified with matrix-assisted laser desorption ionization—time of flight mass spectroscopy (Bruker biotyper, Massachusetts, U.S.). The 60 isolates were categorized as *Candida albicans* (*n* = 11), *Candidozyma auris* (*n *= 10), *C. glabrata* (*n* = 11), *C. parapsilosis* (*n* = 11), *C. tropicalis* (*n* = 10), *C. vulturna* (*n* = 1) and *Cr. neoformans* (*n* = 6). An antifungal susceptibility test was performed for each strains using the Sensititre YeastOne ITAMYUCC (Thermo Fisher Scientifics, Massachusetts, USA) plate according to the manufacturer’s instructions and to The Clinical and Laboratory Standards Institute (CLSI) international guidelines (CLSI M27 2017). The tested antimycotics were: anidulafungin, micafungin, caspofungin, isavuconazole, posaconazole, voriconazole, itraconazole, fluconazole, and amphotericin B. The summarized antimycotic profiles of tested isolates are present in Supplementary Table 1. For planktonic metabolic investigations and growth curves, a representative resistant and susceptible strain was selected from the collection. In *C. albicans, C. glabrata, C. tropicalis, C. parapsilosis* and *Cr. neoformans* the definition was applied to Fluoconazole, based on CLSI breakpoints and modal MICs for *Cr. neoformans* (Espinel-Ingroff et al. 2012). In *C. auris* the definition was applied to Micafungin based on CLSI breakpoints (CLSI M60 2020). All clinical strains are deposited in an internal strain collection at the Institute of Microbiology of Fondazione Policlinico Universitario Agostino Gemelli IRCCS and are available to other researchers upon reasonable request to the corresponding author to ensure reproducibility.

### Antifungal activity of LPs – minimum inhibitory (MICs) and fungicidal (MFCs) concentrations

The MIC was defined as the lowest concentration of peptides capable of inhibiting (visual evaluation of growth buttons) the growth of the investigated yeast, using a broth microdilution method (BMD) according to CLSI guidelines (CLSI M27 2017; Sasoni et al. 2024).

MIC_90_ was defined as the lowest concentration of the peptides at which ≥ 90% of the strains within a test population (same species) were inhibited and showed no visible growth compared to the negative control (cells were grown without peptides).

Also, Minimum Fungicidal Concentration (MFC) has been determined. Briefly, 5 µl of the content of each well was seeded on BCG growth medium agar; after 24 h of incubation, MFCs values were evaluated. MFC_90_ is defined as the lowest concentration at which ≥ 90% of the strains within a test population were killed.

### Kinetic growth curves

The conducted growth curves represent a spectrophotometric investigation in which the increasing optical density (OD) of microorganisms in culture media is linked to their proliferation. To evaluate the differential growth inhibition of the Myr-T4 and Cap-T4 on a sensible and resistant representative strain of each species, a suspension of 0.5 McFarland obtained with Densicheck plus (bioMérieux, Marcy l’Étoile, France) was diluted until the concentration of ~ 10^3^ CFU/mL was obtained in Sabouraud dextrose broth (Merck, Darmstadt, Germany). The suspension was incubated in 96 multi-well plates with a flat bottom (Falcon Corning Incorporated, New York, USA) containing the peptides at different concentrations, ranging from 4 to 64 µg/mL. Untreated cells were used as control. The plates were then incubated at 37 °C, 5% CO_2_ in orbital continuous shaking (250 rpm) inside a Cytation 5 Cell Imaging Multi-Mode Reader (Agilent, Biotek Instrument, VT, USA) for 24 h, monitoring OD changes every 30 min. The optical density was measured at ʎ = 630 nm. The data were subsequently processed and visualized using GraphPad Prism software version 10.4.0 (La Jolla, CA, United States).

### Metabolic evaluation on planktonic yeasts

A 0.5 McFarland suspension of two representative strains per species, one sensitive and one resistant, was diluted to a final concentration of ~ 10^3^ CFU/mL in RPMI 1640 Medium with MOPS and Glucose (Bruker Daltonics, Bremen, Germany). The suspensions were added to 96 multi-well flat-bottom plates (Falcon Corning Incorporated, New York, USA) with Myr-T4 or Cap-T4 at concentrations ranging from 64 to 8 µg/mL. Untreated cells were used as controls and medium alone as blank. Plates were incubated at 37 °C for 24 h. Cellular viability was then assessed using the XTT (2,3-bis-(2-methoxy-4-nitro-5-sulfophenyl)−2H-tetrazolium-5-carboxanilide) assay, using the CyQUANT™ XTT Cell Viability Assay kit (Invitrogen, Thermo Fisher Scientific, Massachusetts, USA) according to the manufacturer’s instructions. The optical density (OD) of the solution in each well was determined with the Cytation 5 Cell Imaging Multi-Mode Reader (Agilent, Biotek Instrument, VT, USA) at a wavelength of 450 and 660 nm. The specific absorbance and cellular viability were determined according to formulas (5) and (6).

5 $$Specific Absorbance=\left[{Abs}_{450 nm Test group}- {Abs}_{450 nm Negative control}\right]- {Abs}_{660 nm Test group}$$

6 $$\mathrm{\%} Cell Viability=(\frac{{OD }_{Test group}}{{OD}_{Negative control}} ) \times 100$$

### Biofilm formation

The enquired strains were tested for their capabilities to form biofilms in a 72 h incubation of ~ 10^5^ CFU/mL in Brain Heart Infusion (BHI) broth (Merck, Darmstadt, Germany) on 96-well plastic flat bottom (Falcon Corning Incorporated, NY, USA) using Crystal violet quantification assay (Thermo Fisher, Waltham, MA, USA), a useful tool to analytically screen the biofilm formation potential on abiotic surfaces of a collection of fungal isolates (Squitieri et al. 2025). Before testing, biofilms were washed three times with PBS to detach non-adherent cells. A representative high biofilm-producing strain was selected for *Candida/Candidozyma* species *C. albicans*, *C. tropicalis*, *C. parapsilosis*, and *C. auris* based on OD outputs, to conduct further analysis of antibiofilm activities of the LPs.

### Metabolic evaluation on biofilm

To evaluate metabolic alterations, 72-h fungal biofilms obtained as described previously were treated with 1 × MIC_90_ and 5 × MIC_90_ concentrations of the LPs in PBS and incubated for 24 h at 37 °C; growth controls were incubated in PBS without the LPs. Biofilm metabolic activity and linked viability were then assessed using the CyQUANT™ XTT Cell Viability Assay (Thermo Fisher Scientific). The OD, specific absorbance, and cellular viability were determined and analyzed according to the manufacturer’s instructions as described for planktonic cells.

### Scanning electron microscopy (SEM)

Biofilms were grown following the previously described protocol, with the adaptation of using 24-well plates (Corning, NY, USA) and thermanox plastic coverslips (Thermo Fisher Scientific) positioned at the bottom of each well; the working volume was adjusted to 1 mL per well. To assess the antibiofilm activity of the two LPs, mature biofilms were exposed to a concentration corresponding to 5 × MIC of each compound, prepared in PBS, and incubated for 24 h at 37 °C. Untreated biofilms were maintained under the same conditions and served as negative controls throughout the experiment. Following treatment, all samples were washed three times with PBS to remove non-adherent cells and residual compounds. Biofilms were then chemically fixed using a 2.5% glutaraldehyde solution (Merck) and subsequently dehydrated through a graded ethanol series, ranging from 30 to 100%, with each step lasting 10 min. Prior to imaging, samples were sputter-coated with gold using a High-Resolution Sputter Coater AGB7234 (Agar Scientific, Stansted, UK) to ensure adequate conductivity. The morphology of fungal cells was examined by scanning electron microscopy using a Supra25 instrument (Zeiss, Oberkochen, Germany); representative micrographs were acquired in secondary electron mode at an accelerating voltage of 5 kV.

### Live/dead fluorescent assay

To assess the cytocidal effect of LPs against selected high biofilm forming *Candida* spp. isolates, Filmtracer™ LIVE/DEAD™ Biofilm Viability Kit (Thermo Fisher Scientific) was conducted. Biofilm was formed as described above in fluorescence-dedicated black 96-well with flat bottom (Ibidi, Fitchburg, WI, USA), washed three times, and treated with 5X MIC concentration of the LPs in PBS for 24 h at 37 °C. The LIVE/DEAD kit was used according to manufacturer’s instructions. Fluorescent microscopy was carried out with a Cytation 5 Cell Imaging Multi-Mode Reader. The excitation wavelengths used were 469 nm and 586 nm while emission wavelengths used were at 525 nm and 647 nm for green (Syto9 emission) and red (Propidium Iodide emission) channels, respectively.

### Hemolytic activity

The peptide’s hemolytic activity was determined at different peptide concentrations based on MICs and MFCs results. Specifically, we used a range from 5 to 100 μM for Trem-4 and from 1 to 50 μM for Myr-T4 and Cap-T4. To perform the test rabbit erythrocytes purified and maintained in Alsever's solution were employed.

For each tested peptide 100 μL of erythrocyte solution at a concentration of 5 × 10^5^ cells/well was incubated for 2 h at 37 C°. At the end of incubation time, the erythrocytes were removed by centrifugation, and the absorbance (Abs) of the supernatant was measured at 490 nm. The negative control consists of erythrocytes in PBS buffer and the positive control of red rabbit cells added with 2% (v/v) Triton X-100.

The hemolysis percentage was calculated as follows (10) (Chen et al. 2023):

10 $$\% Hemolysis=\frac{({Abs }_{Test group}- {Abs }_{Negative control })}{({Abs }_{Positive control}- {Abs }_{Negative control }} \times 100$$

### In vitro cytotoxic activity on fb789 fibroblasts

The cytotoxicity of Trem-4, Cap-T4, and Myr-T4 peptides was tested on primary human FB789 fibroblasts using the MMT (3-(4,5-dimethylthiazol-2-yl)−2,5-diphenyl-2H-tetrazolium bromide) assay.

The cells were grown in Dulbecco’s Modified Eagle Medium (DMEM) containing 10% fetal calf serum, penicillin, and streptomycin antibiotics at 37 °C and in a humidified atmosphere with 5% CO_2_.

Briefly, cells were seeded on 96-well microplates at a density of 1,5X10^4^ cells per well in 100 μL of the medium overnight. Then, the three different peptides were added to the cells, and the cytotoxic effects were evaluated after 3 and 24 h of treatment.

Specifically, after the treatment, the culture medium was removed and replaced with fresh medium containing 0.5 mg/mL MTT (3-(4,5-dimethylthiazol-2-yl)−2,5-diphenyl-2H-tetrazolium bromide) and then incubated at 37 °C in a 5% CO_2_ atmosphere for 3 h. After this time, the supernatant was removed and the formed formazan crystals were solubilized by adding 100 μL of DMSO. After further incubation of 15 min at 37 °C and in an atmosphere of 5% CO_2_, the absorbance was measured at 595 nm using the Epoch2 spectrophotometer (Agilent).

For the negative control, cells were incubated in the presence of only DMEM, while for the positive control NaN3 solution at 3% (v/v) was added (Imperlini et al. 2024). Cell viability was expressed as the percentage of viable cells in the presence of different peptide concentrations compared to the negative control.

The percentage of cell viability was determined as follows (11):

11 $$\% Cells viability= \frac{{\mathrm{A}\mathrm{b}\mathrm{s}}_{\mathrm{T}\mathrm{e}\mathrm{s}\mathrm{t} \mathrm{g}\mathrm{r}\mathrm{o}\mathrm{u}\mathrm{p}}}{{\mathrm{A}\mathrm{b}\mathrm{s}}_{ \mathrm{N}\mathrm{e}\mathrm{g}\mathrm{a}\mathrm{t}\mathrm{i}\mathrm{v}\mathrm{e} \mathrm{c}\mathrm{o}\mathrm{n}\mathrm{t}\mathrm{r}\mathrm{o}\mathrm{l}}} \times 100$$

Additional cytotoxicity experiments on murine 3T3 fibroblasts using the XTT assay are provided in the Supplementary Materials (SM1).

### In vivo toxicity on *Galleria mellonella* model

The toxicity of Myr-T4 and Cap-T4 was evaluated through in vivo studies on *Galleria mellonella* larvae. Scalar dilutions ranging from 64 to 8 ug/mL were tested. Ten microliters of each concentration were injected into the hemocoel in PBS buffer through the last left proleg. Ten larvae were selected randomly for each group; syringes of 0.5 mL without dead volume were used for the injections. Before the treatment, the area was disinfected with 70% ethanol. The larvae were incubated at 30 °C for 3 days; mortality was recorded daily. The vitality of larvae was visually evaluated every 24 h for 3 days; lack of mobility after stimulation and melanization were considered as death indicators according to Loh et al. criteria (Loh et al. 2013). Cocoon formation was excluded as criteria because the phenomenon was not observed in the 3 days of observational period.

### Statistical analysis

All experiments were repeated at least in triplicate to ensure reproducibility. Statistical significance was assessed with Two-way ANOVA with Dunnett's multiple comparison test for all metabolic and cytotoxicity evaluations. Significant difference was defined as *p < 0.05 and ≥ 0.01, **p < 0.01 and ≥ 0.001, ***p < 0.001 and ≥ 0.0001, ****p < 0.0001. Statistical analysis was performed using GraphPad Prism Software version 11.0.0 (San Diego, CA, USA).

## Results

### Steady-state fluorescence measurements and partition constant determination

Tryptophan fluorescence is sensitive to the environment’s polarity. Thus, tryptophan present in the sequence of the three peptides was used as a probe to study and quantify their interaction with membrane mimetic systems.

**Fig. 1:**
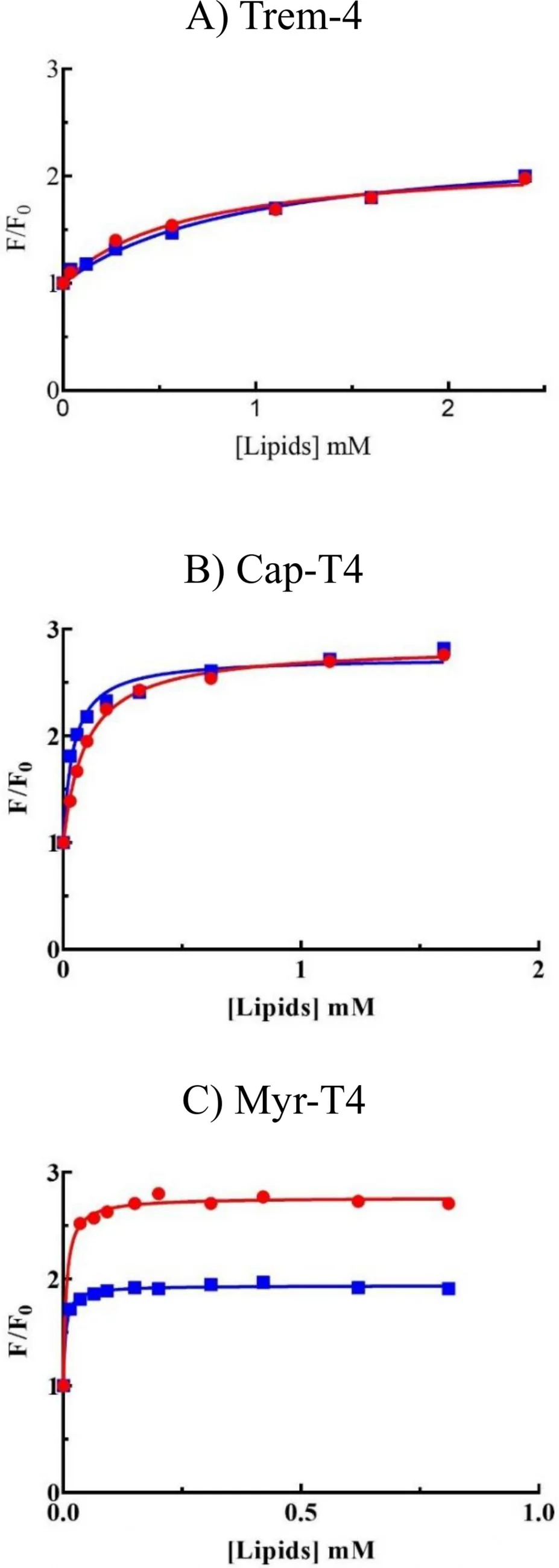
Partition isotherms for Trem-4 (A), Cap-T4 (B), and Myr-T4 (C) peptides in the presence of zwitterionic (red) and anionic (blue) membrane-mimicking systems

Figure 1 shows the binding isotherms for the titration of myristoylated, caprylate, and non-lipidated peptides with POPC and POPC-POPG liposomes, while Table 2 reports the mole fraction partition constant *K*_*x*_, the free energy of partition, and the selectivity ratio.

**Table 2:** Partition constant, selectivity ratio, and free energy change of partition for the peptides Trem-4, Cap-T4 and Myr-T4 in the presence of POPC and POPC-POPG LUVs

**Peptide**	**System**	**K**_**X**_	**ΔG (kJ/mol)**	**Selectivity ratio**
Trem-4	POPC POPC-POPG	(0.88 0 ± 0.19) × 10^5^ (0.57 ± 0.11) × 10^5^	−28.00 −27.00	0.65
Cap-T4	POPC POPC-POPG	(5.8 ± 0.3) × 10^5^ (13.1 ± 0.21) × 10^5^	−32.00 −35.00	2.25
Myr-T4	POPC POPC-POPG	(91.3 ± 18.1) × 10^5^ (119.5 ± 14.6) × 10^5^	−39.00 −40.00	1.31

Partitioning data suggest that lipidated peptides interact with liposomes at higher affinity with respect to Trem-4. The mole fraction partition coefficients, K_x_, of Myr-T4 and Cap-T4 are, respectively, at least one and two orders of magnitude higher compared to the value obtained for Trem-4. Additionally, the lower value of Trem-4 partition free energy, about −27 kJ/mol, suggests poor binding to anionic and zwitterionic membranes.

Furthermore, as evidenced by the selectivity ratio values, lipidation not only enhances the interaction with membranes, but also increases the selectivity toward anionic membranes. Specifically, the Cap-T4 peptide showed the highest observed value, 2.25 (see Table 2). These results are consistent with the findings of our precedent work (Bugli et al. 2022), which reported that myristylation of three short peptides increases their partition constant (K_X_) by one to two orders of magnitude compared with the corresponding non-lipidated peptides.

Moreover, Lin D. and Grossfield A. suggest that the dominant contribution in the interaction with LUVs arises from the insertion of the fatty acid moiety into the lipid bilayer, whereas the positively charged peptide portion provides an additional contribution to binding affinity, particularly in interactions with anionic membranes (Lin and Grossfield 2014).

### Acrylamide quenching experiments

Fluorescence quenching measurements allow us to evaluate the degree of tryptophan accessibility in buffer and in the presence of the membrane-mimicking systems to further clarify the nature of peptide-membrane interactions.

The experimental data were analyzed by linear fit using the Stern–Volmer equation (Eq. 4), plotting F_0_/F as a function of the acrylamide concentration relative to each addition. Table 3 shows the values of the Stern–Volmer constants (K_SV_), the Net Accessibility Factor values, and the percentage of quenching, calculated in POPC and POPG relative to buffer measurements.

**Table 3:** Stern–Volmer constant (K_sv_), percentage of quenching, and NAF values measured in buffer, POPC, and POPC-POPG

Peptide	System	K_sv_ (M^−1^)	NAF	% Quenching
Trem-4	Buffer POPC POPC-POPG	20.2 ± 0.9 11.4 ± 1.0 7.6 ± 0.3	1.00 0.55 0.38	56.4 37.6
Cap-T4	Buffer POPC POPC-POPG	28 ± 0.95 3.9 ± 0.06 2.6 ± 0.08	1.00 0.14 0.07	14.0 9.28
Myr-T4	Buffer POPC POPC-POPG	11.9 ± 0.3 3.2 ± 0.1 3.7 ± 0.2	1.00 0.27 0.31	27.9 31.1

The reciprocal of the K_sv_ value corresponds to the quencher concentration required to reduce the initial fluorescence by 50%. In the presence of LUVs, the decrease of the constant, observed for all analyzed peptides, reflects a reduced quenching efficiency due to a lower exposure of tryptophan to the solvent and, consequently, due to the establishment of more interactions with the lipidic phase.

Interestingly, the quenching percentage of the lipidated peptides in the presence of POPC and POPC–POPG decreases markedly compared to the corresponding values observed for Trem-4 in POPC vesicles (quenching drops from 56.4% for Trem-4 to 14% for Cap-T4 and 27.9% for Myr-T4). Similarly, in POPC–POPG vesicles, it decreases from 37.6% to 9.28% for Cap-T4 and 31.1% for Myr-T4. This reduction indicates that the tryptophan residues within the sequences of the two lipidated peptides are well shielded from the solvent, because of strong interactions between the peptides and the liposomes due to the presence of the acyl chain. These findings confirm what was observed with the partition measurement.

Considering NAF values, low values suggest protection of the fluorophore from the quencher and strong interaction with lipids. The NAF values reported in Table 3 are lower for lipidated peptides than for Trem-4, suggesting, as already found, better interaction with lipid vesicles. Specifically, the NAF value of the Cap-T4 peptide observed in the presence of POPC-POPG (0.07) indicates a strong preference for anionic lipid vesicles, as observed for the selectivity ratio obtained with the partition measurements.

### CAC determination

Lipopeptides typically assemble into supramolecular structures. The self-assembly behavior of lipidated peptides was assessed by determining the critical aggregation concentration (CAC), which corresponds to the concentration at which monomers spontaneously associate in aqueous solutions (Bugli et al. 2022). This was investigated using 8-anilino-1-naphthalenesulfonic acid (ANS), a fluorescent probe whose emission intensity increases in apolar environments.

**Fig. 2:**
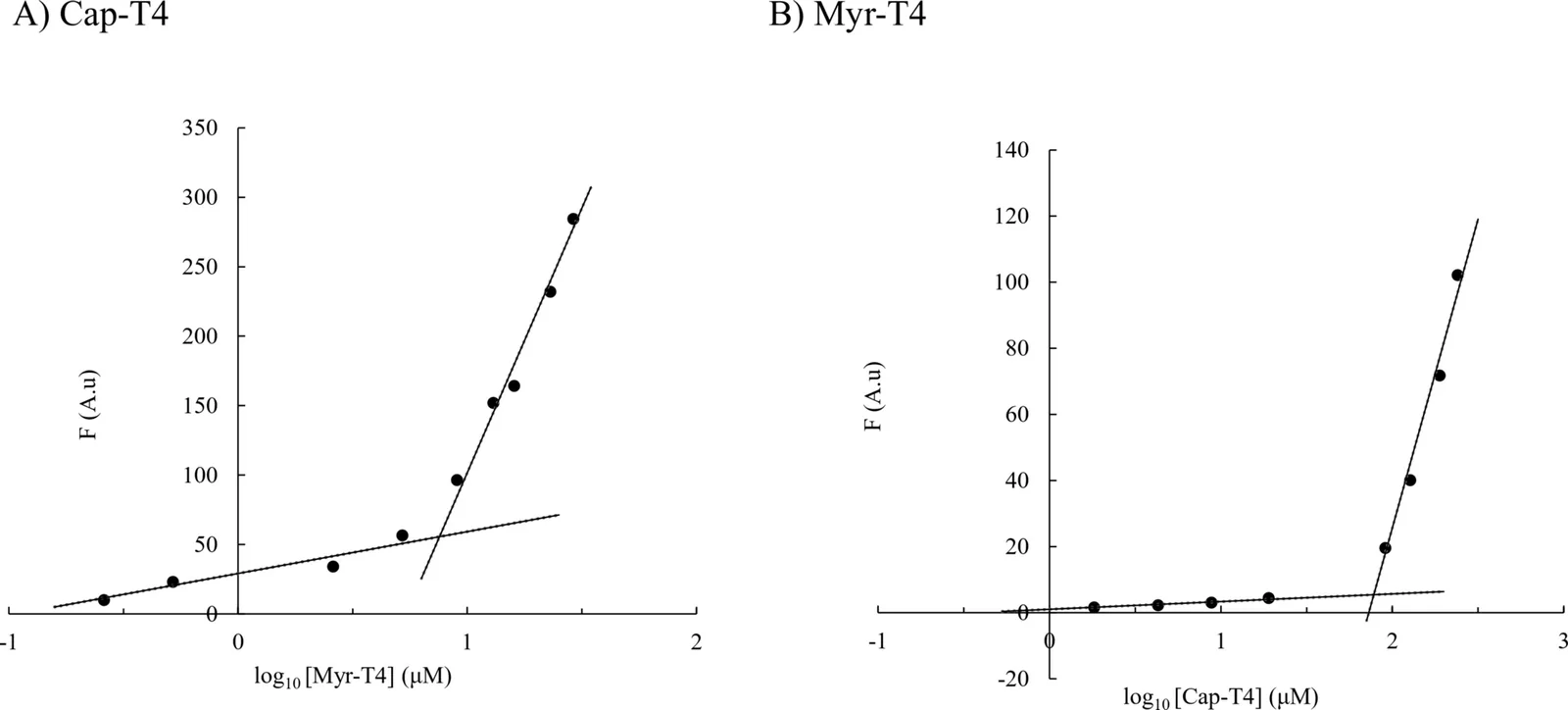
Plot of ANS fluorescence at 460 nm as a function of the logarithm of Cap-T4 and Myr-T4 peptides. The intersection of the lines indicates the CAC

Specifically, ANS fluorescence was monitored as a function of increasing concentrations of the tested lipopeptide. Plotting the fluorescence intensity (values were observed at 460 nm) against the logarithm of the lipopeptide concentration (Fig. 2), two different line trends were obtained: at low concentrations, the fluorescence intensity increased slowly until reaching a threshold, after which a sharp increase in fluorescence was observed. This behavior indicates a change in the state of the lipopeptides’ aggregation. For each peptide, the CAC was determined as the intersection point of the two lines.

From the analysis of the experimental data, the CAC results (74 ± 5) μM for Cap-T4 and (7.4 ± 0.3) μM for Myr-T4. The lower CAC value observed for Myr-T4 was a consistent value determined in our precedent work for the peptides Myr-A (Myr-WFGKLYRGITK), Myr-B (Myr-WRGITKVVKKV), and Myr-C (Myr-WVVKKVKGLLK) and depends on the length of the acyl chain used for lipidation of Trem-4 (Bugli et al. 2022). In fact, the critical aggregation concentration of lipopeptides decreases with the increase of the acyl chain length (Sikorska et al. 2018).

### Circular dichroism

**Fig. 3:**
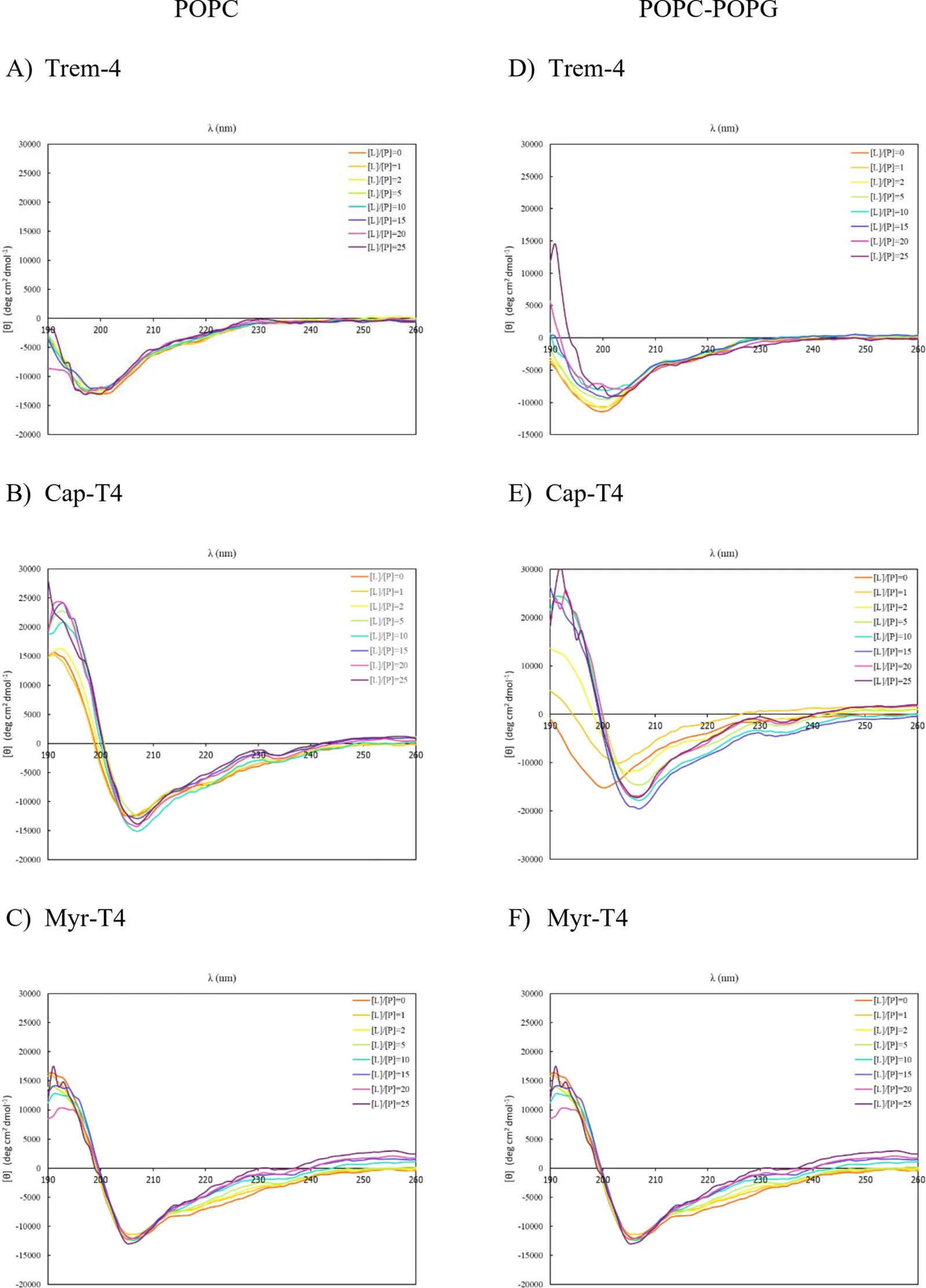
CD spectra of Trem-4 (**A–D**), Cap-T4 (**B–E**) and Myr-T4 (**C–F**) peptides in phosphate buffer (red) and in the presence of increasing amounts of lipid vesicles

Structural transitions of the peptides upon addition of lipid vesicles were investigated by circular dichroism (CD) spectroscopy. We examined the conformational changes of both lipidated and non-lipidated peptides following their interaction with anionic and zwitterionic membrane-mimicking systems. Figure 3 shows representative titration curves for all peptides recorded at increasing lipid vesicle concentrations. The peptide-to-lipid ratios explored ranged from 1:0 to 1:25.

As can be seen from the spectra reported in Fig. 3, the Trem-4 peptide adopts a random coil conformation in buffer, as indicated by a strong negative band at ~ 200 nm (red line). This conformation appears to persist even after the addition of lipid vesicles.

For the Cap-T4 and Myr-T4 peptides, the spectrum reveals a strong positive band at ~ 195 nm, accompanied by two weak ones at ~ 208 nm and ~ 222 nm. This suggests that the addition of the acyl chain induces a small transition towards an α-helical conformation. This structure is further stabilized upon the addition of LUVs, especially for Cap-T4 in the presence of anionic vesicles (POPC-POPG).

The impact of the acyl chain on peptide structure is in agreement with data reported by Rounds T. and Straus SK., who stated that acylated peptides with 8, 10, or 12 carbon atoms display a more structured conformation than the corresponding non-lipidated peptides, particularly in the presence of membrane-mimetic systems (Rounds and Straus 2020).

### Antifungal activity of LPs

Overall, the clinical isolates displayed susceptibility profiles consistent with their known intrinsic and acquired resistance traits. High susceptibility to echinocandins was observed among *C. albicans*, *C. tropicalis* and *C. parapsilosis*, while reduced susceptibility to azoles—particularly fluconazole and voriconazole—was detected in subsets of these species.

As expected, *C. glabrata* exhibited elevated fluconazole MICs, while *C. auris* showed universal resistance to fluconazole (100%) and variable resistance to echinocandins and azoles. *Cr. neoformans* isolates were uniformly resistant to echinocandins, but remained susceptible to azoles and amphotericin B. These susceptibility profiles, shown in Tables S1, confirm the clinical relevance and heterogeneity of the strain collection and provide an appropriate reference framework for the evaluation of the antifungal activity of the lipidated peptides.

Trem-4 when tested against *Candida* spp. and *Cr. neoformans*, showed MIC values above 64 µg/mL (Table S2).

The MIC and MFC values distributions for Cap-T4 and Myr-T4 against investigated clinical fungal species are summarized in Table 4.

**Table 4:** MIC and MFC values distribution of Cap-T4, and Myr-T4, reported in μg/mL as MIC90, MFC90, median value and range against different fungal isolates (*n* = 60)

Species (number of isolates)	Cap-T4 MIC₉₀	Cap-T4 median MIC (range)	Myr-T4 MIC₉₀	Myr-T4 median MIC (range)	Cap-T4 MFC₉₀	Cap-T4 median MFC (range)	Myr-T4 MFC₉₀	Myr-T4 median MFC (range)
*C. albicans* (11)	32	32 (16–32)	32	32 (32–64)	32	32 (32–32)	> 64	32 (32– > 64)
*C. glabrata* (11)	32	32 (16–32)	32	32 (16–32)	32	32 (16–64)	32	32 (32–32)
*C. tropicalis* (10)	16	8 (8–16)	32	32 (32–32)	32	16 (16–32)	64	32 (32–64)
*C. parapsilosis* (11)	64	32 (16–64)	32	32 (32–32)	64	64 (16–64)	> 64	> 64 (32– > 64)
*C. auris* (10)	64	64 (16–64)	32	32 (16–32)	> 64	> 64 (32– > 64)	> 64	64 (32– > 64)
*Cr. neoformans* (6)	16	8 (8–16)	16	16 (16–16)	16	16 (16–32)	32	32 (32–32)
*C. vulturna* (1)	64	64 (ND)	64	64 (ND)	64	64 (ND)	64	64 (ND)

Both lipidated peptides exhibited markedly enhanced antifungal activity compared with the non-lipidated parent peptide Trem-4, which was therefore excluded from further comparative analyses.

Among the lipidated analogues, Cap-T4 showed the most consistent inhibitory activity across the different yeast species, with median MIC values as low as 8 µg/ml against *C. tropicalis* and *Cr. neoformans*. In contrast, Myr-T4 displayed a more heterogeneous activity profile but demonstrated lower median MIC and MFC values against the clinically relevant pathogen *C. auris*, while maintaining a broad spectrum of activity across the tested species.

While lipidation clearly enhanced the inhibitory activity of both lipopeptides, improvements in fungicidal activity were not uniformly observed across all species. Specifically, Cap‑T4 displayed median MFC values above 64 µg/ml against *C. auris*, whereas Myr‑T4 showed elevated MFC values against *C. parapsilosis*.

### Kinetic growth curves

**Fig. 4:**
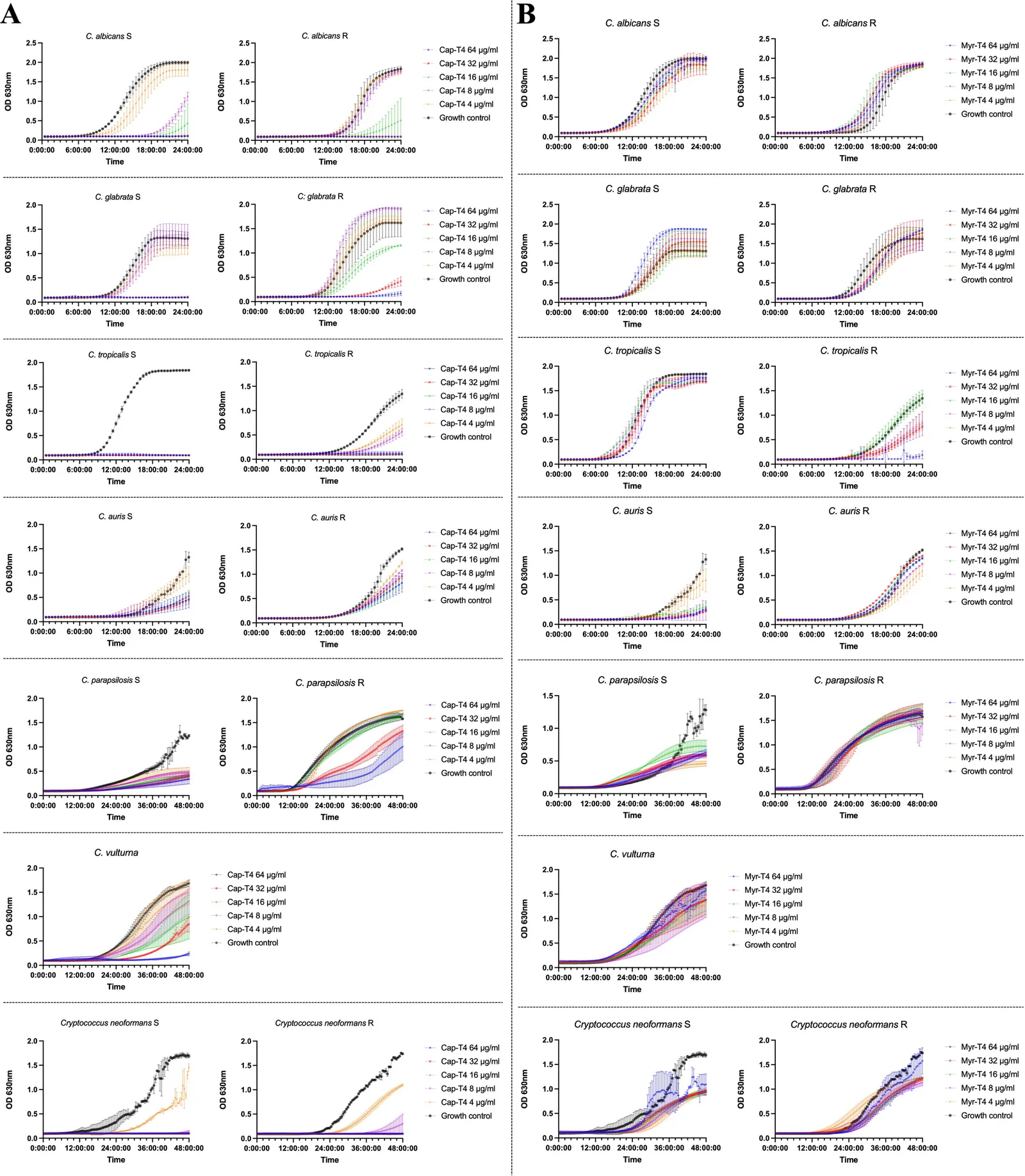
Growth kinetics of fungal strains in Sabouraud medium in the presence of Cap-T4 (**A**) or Myr-T4 (**B**). Optical density (OD₆₃₀ nm) was monitored over time (24 h or 48 h when appropriate) for susceptible (S) and resistant (R) strains of *C. albicans*, *C. glabrata*, *C. tropicalis*, *C. auris*, *C. parapsilosis*, *C. vulturna*, and *Cr. neoformans*. Compounds were tested at 64, 32, 16, 8, and 4 μg/mL; untreated cultures served as growth controls. Data are presented as mean ± SD of independent replicates

To assess the behavior of the LPs in a nutrient-rich environment, the antifungal activity of Cap-T4 and Myr-T4 at active concentrations was monitored over time in Sabouraud dextrose broth by performing growth-curve analyses. Optical density measured at wavelength equal to 630 nm (OD₆₃₀) was monitored every 30 min for up to 24 h (*C. albicans, C. glabrata, C. tropicalis, C. auris*) or 48 h (*C. parapsilosis, C. vulturna* and *Cr*. *neoformans*) of kinetic incubation in broth. The related growth curves are shown in Fig. 4 and stratified by LP treatment (Fig. 4A for Cap-T4 and Fig. 4B for Myr-T4) and fungal species (lines). Within each panel, sub-columns are organized according to antifungal susceptibility profile, with the azole-susceptible strain (S) displayed on the left and the azole-resistant strain (R) on the right.

In a nutrient-rich medium, Cap-T4 showed a strong dose effect, with near-baseline OD at 64–32 μg/mL in multiple species, while intermediate/partial regrowth emerged only at lower concentrations. For *C. albicans* (S), the 24 h control reached 1.996 ± 0.035, whereas 64 and 32 μg/mL remained low (0.099 ± 0.005 and 0.204 ± 0.006), and 16 μg/mL yielded a partially restored but variable endpoint (0.443 ± 0.489).

A clear “partial inhibition” window was evident for *C. tropicalis* (R), where 64–16 μg/mL stayed close to baseline (24 h OD: 0.112 ± 0.005, 0.099 ± 0.006, 0.109 ± 0.007) versus 1.345 ± 0.088 in the control, while 8 μg/mL allowed intermediate growth (0.558 ± 0.055), consistent with concentration-dependent relief of inhibition.

For *C. auris* (S), growth was reduced at high doses (24 h OD: 0.385 ± 0.006 at 64 μg/mL and 0.564 ± 0.016 at 32 μg/mL vs 1.327 ± 0.104 control), with partial regrowth persisting at 16 μg/mL (0.526 ± 0.120). *Cr. neoformans* (S), inhibition remained essentially complete through 16 μg/mL (48 h OD: 0.103 ± 0.002 at 64; 0.096 ± 0.000 at 32; 0.093 ± 0.001 at 16 vs 1.689 ± 0.038 control), whereas 4 μg/mL supported substantial recovery (1.456 ± 0.415), indicating a threshold-like response rather than a smooth gradient.

In Sabouraud dextrose broth, Myr-T4 exerted overall limited inhibitory activity on *Candida* spp. and *Cr. neoformans* planktonic growth. Azole-susceptible *C. albicans*, *C. glabrata* and *C. tropicalis* showed growth curves largely overlapping the untreated controls; for example, *C. albicans* (S) reached 1.996 ± 0.034 (control) versus 1.988 ± 0.060 at 64 μg/mL, while *C. glabrata* (S) displayed even higher OD values in treated cultures (1.308 ± 0.132 control vs 1.863 ± 0.016 at 64 μg/mL). Corresponding azole-resistant isolates of *C. albicans* and *C. glabrata* likewise showed no consistent concentration-dependent inhibition.

In contrast, *C. tropicalis* (R) and *C. auris* (S) exhibited marked suppression at the highest concentration, with OD values decreasing from 1.345 ± 0.088 to 0.190 ± 0.084 and from 1.327 ± 0.104 to 0.317 ± 0.036, respectively, at 64 μg/mL. At 48 h, *C. parapsilosis* (S) and *Cr. neoformans* (S) showed reduced but not strictly dose-dependent growth (1.281 ± 0.083 vs 0.631 ± 0.045 and 1.689 ± 0.038 vs 1.091 ± 0.216 at 64 μg/mL). Overall, Myr-T4 induced modest, strain-dependent effects in Sabouraud medium, with clear concentration-associated inhibition mainly in *C. tropicalis* (R) and *C. auris* (S).

### Metabolic evaluation on planktonic yeasts

For all tested species, exposure to Cap-T4 and Myr-T4 resulted in a concentration-dependent reduction of metabolic activity, as assessed by XTT reduction after 24 h (Figure S1). The extent of metabolic inhibition correlated well with the MIC values obtained by broth microdilution, with the most pronounced effects observed at concentrations equal to or above the MIC.

In *C. albicans*, both peptides markedly reduced metabolic activity at the highest concentration in susceptible and resistant isolates, whereas limited effects were observed at sub-MIC concentrations. In *C. glabrata* and *C. tropicalis*, a strong decrease in metabolic activity was detected at 32–64 µg/mL, while partial preservation of metabolic activity was observed at lower concentrations. *C. parapsilosis* isolates showed variable responses, with susceptible strains exhibiting a clearer reduction in metabolic activity than resistant ones.

In *C. auris*, high peptide concentrations were required to significantly impair metabolic activity, whereas lower concentrations had minimal impact. *Cr. neoformans* displayed a marked reduction in metabolic activity at higher peptide concentrations for both compounds, and a similar trend was observed for the single *C. vulturna* isolate. Overall, the metabolic profiles at 24 h were consistent with the susceptibility patterns observed in the MIC assays.

### Metabolic and morphological evaluation on biofilm

XTT assays performed on 72 h mature biofilms showed a peptide- and species-dependent reduction of metabolic activity, expressed as percentage relative to untreated controls. For Myr-T4, biofilms of *C. tropicalis*, *C. parapsilosis*, and *C. auris* exhibited a clear decrease in metabolic activity already at 1 × MIC, with values ranging approximately between 28% and 50% of the control. At 5 × MIC, metabolic activity was further reduced, reaching very low or near-zero values in *C. albicans* and *C. parapsilosis*, while remaining consistently low in *C. tropicalis* and *C. auris*. In contrast, *C. albicans* showed a higher variability at 1 × MIC, with metabolic activity comparable to or slightly exceeding control levels, followed by a pronounced reduction at 5 × MIC.

For Cap-T4, the reduction in metabolic activity was overall more heterogeneous across species and concentrations. *C. tropicalis* and *C. parapsilosis* showed moderate decreases at 1 × MIC, with a more evident reduction at 5 × MIC, although residual metabolic activity was still detectable. In *C. albicans*, metabolic activity was reduced at 1 × MIC and almost completely suppressed at 5 × MIC. Notably, *C. auris* displayed marked variability between replicates, with one duplicate showing strong inhibition at 1 × MIC and an unexpected increase in metabolic signal at 5 × MIC.

**Fig. 5:**
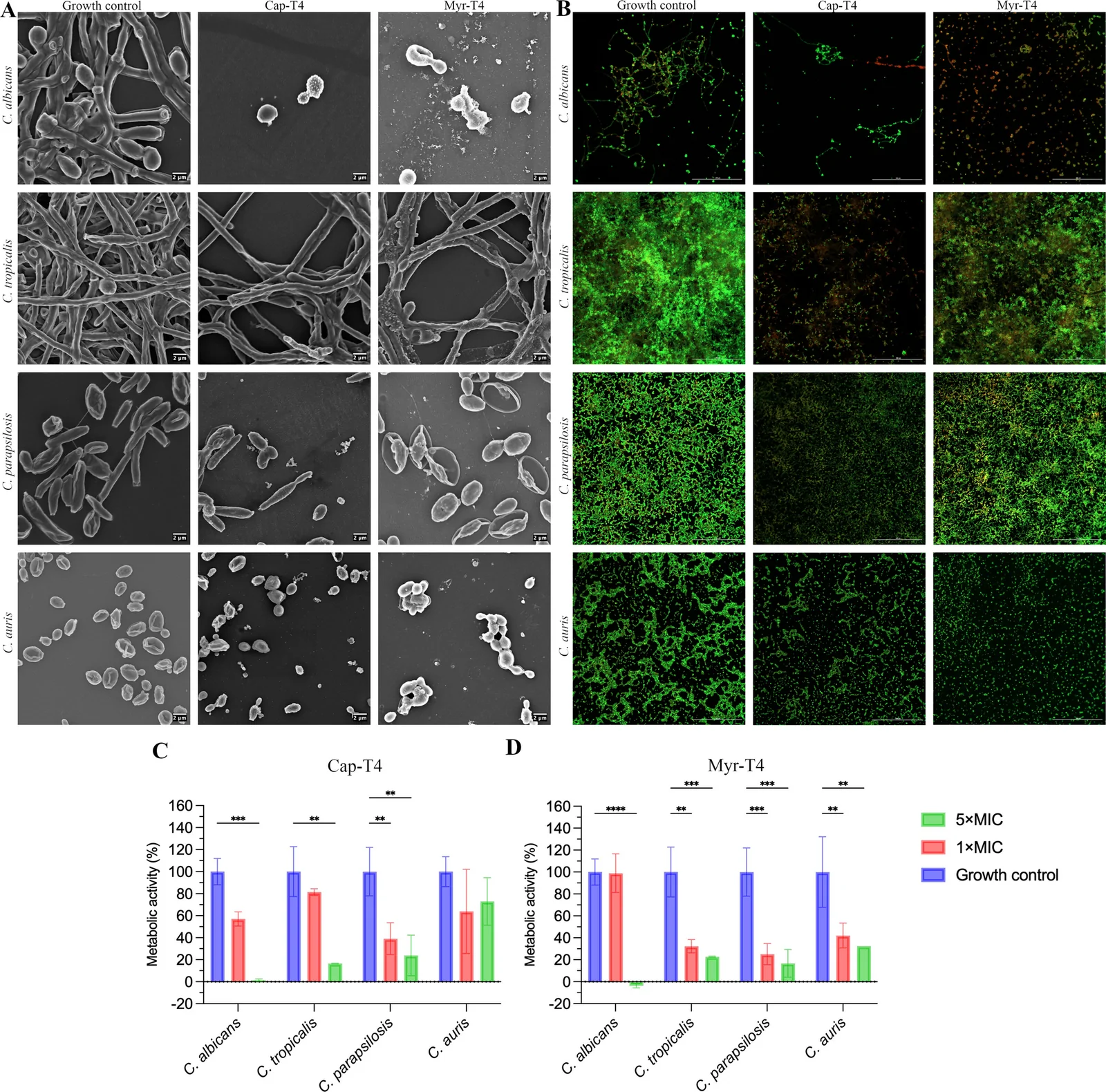
Evaluation of antibiofilm activity of Cap-T4 and Myr-T4 by SEM, Live/Dead staining, and XTT assay. Mature 72-h biofilms were treated with the tested peptides at concentrations corresponding to 5 × MIC concentrations (also 1 × MIC for XTT assay) of Cap-T4 and Myr-T4. (**A**) Structural alterations of the biofilm architecture were visualized by scanning electron microscopy (SEM). (**B**) Biofilm viability was further assessed with confocal microscopy using Live/Dead staining, distinguishing metabolically active cells (green, Syto9 emission) from membrane-compromised cells (red, Propidium iodide emission). (**C–D**) Metabolic activity of treated biofilms with 1 × MIC and 5 × MIC (C) Cap-T4 or (D) Myr-T4 was quantified by XTT reduction assay. Untreated biofilms served as controls. Data are normalized to controls and reported as mean ± SD. Statistical significance was assessed relative to the control condition; *p < 0.05 and ≥ 0.01, **p < 0.01 and ≥ 0.001, ***p < 0.001 and ≥ 0.0001, ****p < 0.0001

As evident in Fig. 5, SEM analysis revealed marked peptide‑induced alterations in biofilm architecture compared to untreated controls. Control biofilms exhibited a dense and highly organized structure, characterized by compactly packed yeast cells embedded within an extensive extracellular matrix. In contrast, biofilms exposed to either lipopeptide displayed a pronounced loss of structural integrity, with evident biofilm thinning, reduced intercellular cohesion and partial disruption of the extracellular polymeric substance. These alterations resulted in sparsely distributed and more exposed fungal cells across the biofilm surface.

Live/Dead fluorescence staining for *C. tropicalis* corroborated the SEM findings, revealing a substantial increase in red-fluorescent cells for biofilms treated with Cap-T4, indicative of compromised membrane integrity. The other treated biofilms do not show an evident imbalance in the red/green fluorescent emission homeostasis, but a massive reduction in biofilm numerosity and thickness is evident in all samples. Based on microscopy data, *C. parapsilosis* biofilm seems to be the least affected by peptides’ treatment, while XTT assay clearly indicates that a 5 × MIC of both LPs statistically reduces the metabolic activity and overall viability of cells.

### In vitro and in vivo biocompatibility evaluation

The cytotoxicity of Trem-4 and its lipidated analogues, Cap-T4 and Myr-T4, was evaluated on mammalian cells using two complementary in vitro models. Short- and long-term cytotoxic effects were assessed on the human fibroblast cell line FB789 by MTT assay after 3 and 24 h of treatment, while cytotoxicity on murine embryonic immortalized 3T3 fibroblasts was evaluated after 24 h using the XTT assay.

**Fig. 6:**
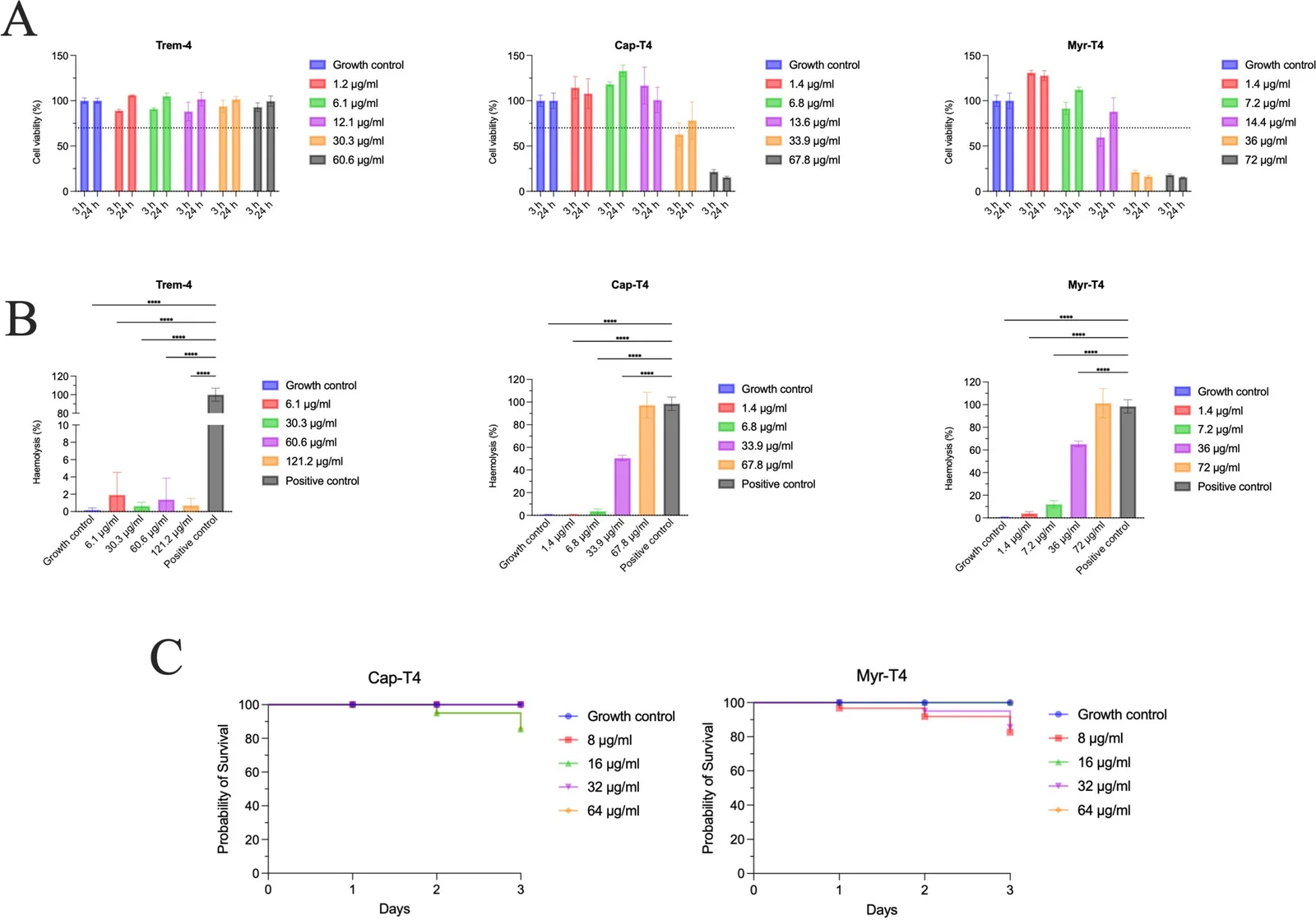
In vitro cytotoxicity and hemolytic activity of Trem-4, Cap-T4, and Myr-T4, and in vivo toxicity of Cap-T4, and Myr-T4. (**A**) In vitro cytotoxicity on human fibroblast FB789 cells after 3 h and 24 h of treatment, evaluated by MTT assay. Data are normalized to untreated controls. The horizontal dotted line indicates the 70% cell viability threshold according to ISO 10993–5:^2009^. (**B**) Hemolytic activity on rabbit erythrocytes, expressed as percentage relative to the positive control (100% hemolysis) after treatment with increasing peptide concentrations. (**C**) In vivo toxicity of Cap-T4, and Myr-T4 in *Galleria mellonella* larvae. Larvae were injected with 64, 32, 16, and 8 µg/mL of each lipopeptide, and survival was monitored at 24, 48, and 72 h. Control larvae were injected with PBS only. Data are presented as mean ± SD. Statistical significance was assessed relative to the control or positive control as appropriate; *p < 0.05 and ≥ 0.01, **p < 0.01 and ≥ 0.001, ***p < 0.001 and ≥ 0.0001, ***p < *0.0001*

As shown in Fig. 6, Trem-4 did not induce cytotoxic effects in either cell line at any of the tested concentrations. In contrast, the lipidated peptides exhibited concentration-dependent cytotoxicity. In FB789 cells (Fig. 6, panel A), Myr-T4 showed marked toxicity, with cell viability decreasing to approximately 20% at 36 µg/ml (25 µM), whereas Cap-T4 affects less cell viability at a similar concentration (33.9 µg/ml; 25 µM). However, Cap-T4 toxicity increased at higher concentrations, reaching approximately 80% cell death at 67.8 µg/ml (50 µM). No significant differences were observed between short-term (3 h) and long-term (24 h) treatments for either lipidated peptide. Consistently, cytotoxicity assessment on 3T3 fibroblasts (Figure S2) revealed that Myr-T4 induced a reduction in cell viability greater than 30% at the highest tested concentrations (64 and 32 µg/mL or 44.4 and 22.2 µM), exceeding the cytotoxicity threshold defined by ISO 10993–5:^2009^. Under the same conditions, Cap-T4 showed cytotoxic effects only at 64 µg/mL (or 47.2 µM). At lower concentrations (16 and 8 µg/mL or 11.1 and 5.6 µM for Myr-T4; and 32, 16, and 8 µg/mL or 23.6, 11.8 and 5.9 µM for Cap-T4), cell viability remained above 70%, indicating the absence of cytotoxicity according to the ISO standard.

Rabbit erythrocytes have been used to investigate the hemolytic effect of the three peptides. Interestingly, as reported in Fig. 6 panel B, peptide Trem-4 did not show a hemolytic effect until 121.2 µg/ml (100 µM), while the percentage of hemolysis induced by lipidated peptide increased at increased concentration of tested peptides. Overall, peptide Myr-T4 displays stronger hemolytic activity, causing ~ 42% hemolysis already at 14.4 µg/ml (10 µM). Under similar conditions (13.6 µg/ml or 10 µM), peptide Cap-T4 induces only ~ 4% hemolysis. However, its hemolytic activity increases to 51% at 33.9 µg/ml (25 µM). At the highest concentration tested, 67.8 μg/ml for Cap-T4 (or 50 μM) and 72 μg/ml for Myr-T4 (or 50 μM), both lipidated peptides cause 100% erythrocyte lysis.

The major hemolysis induced by Myr-T4 can be explained considering the length of the acyl chain used for the lipidation of the peptide sequence. In fact, an increase in hydrophobicity could lead to major toxicity in mammalian cells due to the loss of selectivity for microbial anionic membranes (Húmpola et al. 2017; Greber et al. 2017).

These results are in line with observations reported in the literature for other lipopeptides (Jensen et al. 2020).

Húmpola et al. showed that myristoylation of the decapeptide IKQVKKLFKK caused nearly 80% hemolysis at 50 μM, whereas the corresponding caprylated analogue induced only about 15% erythrocyte lysis at the same concentration (Húmpola et al. 2017).

Figure 6 panel C shows the in vivo toxicity of Cap-T4 and Myr-T4, evaluated using *Galleria mellonella* larvae, with survival monitored at 24, 48 and 72 h after injection of ten microliters containing the peptides at a concentration of 64, 32, 16, and 8 µg/mL. Larval viability remained high across all tested concentrations and time points. At 24, 48, and 72 h survival rates were ≥ 90% and probability of survival ≥ 82.6% in all groups.

## Discussion

The infections caused by pathogenic fungi and invasive fungal diseases have increased significantly over the past few decades, reaching 6.55 million each year with over 3.75 million deaths (Denning 2024). This rise is driven by advances in medical treatments expanding the immunosuppressed population and by antimicrobial resistance (Buda De Cesare et al. 2020). World Health Organization (WHO) in 2022 drew up a list of pathogenic fungi posing major threats to public health, similar to that established for antibiotic-resistant bacteria. The fungi critical group includes *Cr. neoformans* and *Candida* spp., associated with mortality rates up to 88% and highlighting the need for more effective and selective antifungal agents (World Health Organization 2022).

In this context, functionalization of short cationic peptide sequences using N-lipidation can enhance their antimicrobial activity and selectivity against pathogens (Malina and Shai 2005; Lin and Grossfield 2014; Húmpola et al. 2017; Sikorska et al. 2018; Bugli et al. 2022). Moreover, N-terminal lipidation should improve proteolytic and serum stability often through binding to serum albumin (Helmy et al. 2026).

This work, therefore, examines the impact of N-caprylation and N-myristoylation on the antifungal activity and cellular selectivity of a decapeptide named Trem-4, which is derived from the previously studied peptide Trem-HSK (Squitieri et al. 2024).

N-lipidation of peptide sequences increases hydrophobicity and, consequently, promotes self-assembly. In aqueous solution, above the critical aggregation concentration (CAC), monomers self-assemble into organized structures to shield the hydrophobic tail from the polar environment (Bugli et al. 2022). CAC values for Cap-T4 and Myr-T4 were determined using a spectroscopic method with the fluorescence probe 8-anilinonaphthalene-1-sulfonate (ANS). As already documented (Húmpola et al. 2017), CAC values decreased with the chain length increase: Myr-T4 showed a tenfold lower CAC (7.4 ± 0.3) μM, compared to Cap-T4 (75 ± 4) μM.

In addition, lipidation reduced the net positive charge from + 3 to + 2. Therefore, the biological effects observed cannot be attributed solely to enhanced lipophilicity. These two physicochemical changes may have opposing effects on antifungal activity: increased hydrophobicity may favor membrane insertion and disruption, whereas reduced cationic charge may decrease electrostatic attraction toward negatively charged fungal membrane components. Thus, the activity of N-lipidated analogs should be interpreted as the combined effect of altered hydrophobicity, charge and membrane interaction.

To evaluate the membrane interaction, the partitioning of Trem-4, Cap-T4 and Myr-T4 peptides in the presence of POPC and POPC-POPG was evaluated by spectroscopic methods.

Although liposomes do not fully capture the complexity of natural membranes, they have been used to reproduce the charge of fungal anionic membranes and mammalian cell zwitterionic membranes (Bugli et al. 2022).

The partitioning constants obtained from processing experimental data indicate that the N-lipidation determines a significant improvement in the ability of the Trem-4 peptide to interact with the used membrane-mimicking systems.

Indeed, Trem-4 peptide exhibited very low interaction and the absence of selectivity against the membrane models tested. Caprylation of Trem-4, in particular, improved selectivity towards anionic membrane: Cap-T4 showed a high value, 2.25, of selectivity ratio, which represents an important parameter in the development of a potential drug, compared to Trem-4, 0.65, and Myr-T4, 1.31. Conjugation of fatty acids to the synthetic peptide CG117-136 resulted in a higher α-helical content compared to the native peptide in a membrane-mimetic environment, promoting deeper insertion into liposomes and significantly increased its ability to disrupt microbial membranes (Mak et al. 2003).

Quenching fluorescence studies using acrylamide as a collisional quencher supported the partition measurement data. In particular, the Stern–Volmer constant values in buffer solution were higher than those observed in the presence of membrane-mimicking systems, indicating reduced quenching efficiency due to lower solvent exposure of tryptophan inside the sequence of all three peptides and consequently stronger interaction with liposomes. The lowest NAF value, 0.07, was recorded for Cap-T4 in an anionic system, further confirming its higher affinity for anionic liposomes.

To verify biochemical characterization, an extensive clinically relevant microbiological evaluation was performed to assess LPs' efficacy against a broad panel of monocellular fungal pathogens in planktonic and biofilm forms. The comparatively higher inhibitory concentrations observed for Myr-T4 (~ 32 µg/mL or 22.2 µM) may reflect its propensity to self-aggregate at low concentrations in aqueous solution, an effect previously linked to reduced interaction with microbial membranes due to peptide self-assembly. This behavior has been described for other lipidated antimicrobial peptides and may impede effective membrane engagement despite intrinsic (Bellavita et al. 2023). Although both Myr-T4 and Cap-T4 showed relatively high MIC values, potentially limiting potency, their activity remained largely unaffected by conventional resistance phenotypes. Indeed, MIC values remain relatively constant across strains regardless of azole or echinocandin susceptibility.

The MIC values are encouraging when contextualized with existing literature on synthetic lipidated peptides. An example is the decapeptide IKQVKKLFKK lipidated with fatty acids that showed enhanced antifungal activity. In particular, the caprylated and myristoylated derivatives exhibited MICs of ~ 46 µM (≈ 64 µg/mL) and ~ 174 µM (≈ 255 µg/mL), respectively, against *Candida* spp. reference strains (Húmpola et al. 2017).

Other lipidated analogs such as Myr-A (Myr-WFGKLYRGITK), Myr-B (Myr-WRGITKVVKKV), and Myr-C (Myr-WVVKKVKGLLK) exhibited MIC ranges of ~ 8–32 µg/mL (5.2–21.2 µM) against clinical *Candida* isolates, except *C. auris,* which showed marked resistance, confirming that fatty acid acylation of short cationic sequences can significantly enhance antifungal activity (Bugli et al. 2022). Together, these findings further support the concept that increasing the hydrophobic character of short antimicrobial peptide sequences via fatty acid conjugation enhances their antifungal properties, although the specific chain length and peptide context crucially influence potency and spectrum (Húmpola et al. 2017).

Growth-curve profiling largely mirrored RPMI 1640 (2% glucose) static MIC endpoints for Cap‑T4, with near-baseline OD at 32–64 µg/mL and limited regrowth at lower concentrations, consistent with Cap‑T4 median MICs spanning 8–64 µg/mL and a threshold-like inhibition in some species (e.g., suppression through 16 µg/mL in *Cr. neoformans*). By contrast, Myr‑T4 showed modest or absent suppression in Sabouraud dextrose broth, with several *Candida* spp. exhibiting growth curves overlapping controls even at 64 µg/mL, despite RPMI-based median MICs around 32 µg/mL. These discrepancies likely reflect non-equivalence between endpoint MICs measured under static conditions in RPMI and kinetic growth-curve readouts obtained in a nutrient-rich medium.

Hydrodynamics and medium composition can shift antifungal endpoints: shaking improves oxygenation, alters growth kinetics and MIC interpretability in *Cryptococcus* microdilution assays (Rodríguez-Tudela et al. 2000), while RPMI 1640 and Sabouraud dextrose broth differ in composition and glucose content (~ 2 g/L versus ~ 20 g/L, respectively) (Weerasekera et al. 2016). Such differences may alter growth and stress physiology, shifting the inhibitory window observed in kinetic assays (Vahedi-Shahandashti et al. 2023). The stronger medium dependence of Myr‑T4 may relate to its higher hydrophobic load (myristoyl), promoting self-association or non-productive partitioning in complex broth—behaviors, as described for lipidated AMPs (Rounds and Straus 2020).

The metabolic responses at 24 h closely mirrored the broth microdilution MICs, indicating that metabolic inhibition largely reflects growth inhibition under planktonic conditions. In several species, particularly *C. glabrata* and *C. auris*, sub-MIC concentrations were associated with preserved or increased metabolic activity, suggesting a limited peptides’ impact below inhibitory concentrations and highlighting species-specific tolerance mechanisms. Although both lipidated peptides impaired metabolic activity at higher concentrations, metabolic inhibition varied among species, reflecting the distinct antifungal profiles of Myr-T4 and Cap-T4. Mature biofilms displayed markedly reduced susceptibility to both lipidated peptides than planktonic cells, confirming the well-known protective role of the biofilm lifestyle (Kaur and Nobile 2023). Increasing peptide concentration from 1 × to 5 × MIC enhanced metabolic inhibition but failed to achieve complete biofilm eradication, highlighting the intrinsic tolerance of mature biofilms. Differences between Myr-T4 and Cap-T4 were more evident in biofilms, suggesting that lipid chain length may influence peptide penetration or interaction within the biofilm matrix (Batoni et al. 2016). The persistence of metabolic activity after treatment supports the contribution of biofilm-associated mechanisms, including limited diffusion, altered membrane composition, or the presence of metabolically active subpopulations (LaFleur et al. 2006). These findings underscore how subtle structural modifications in LPs can translate into markedly different functional outcomes within complex fungal communities. Microscopy analyses provide important insights into the effects of peptide treatment on the structural organization and viability of mature *Candida* biofilms. SEM observations indicate that both peptides at 5 × MIC severely disrupted biofilm architecture, causing morphological deformations, biofilm thinning, and loss of cellular cohesion. These structural alterations suggest that peptide activity compromises the biofilm physical stability, which is a key determinant of persistence and antimicrobial agents tolerance (Fanning and Mitchell 2012).

Live/Dead fluorescence staining further supports a peptide-induced impairment of biofilm integrity, although membrane damage appears to be species- and peptide-dependent. In *C. tropicalis*, Cap-T4 increased membrane-compromised cells, consistent with a direct membranolytic activity. In contrast, the absence of a pronounced red/green fluorescence imbalance in other treated biofilms suggests that acute membrane permeabilization is not uniformly induced across species or conditions. Despite the limited Live/Dead signal of membrane damage in some species, all treated biofilms showed reduced biofilm thickness and cell density, indicating a loss of biofilm biomass and spatial organization even when overt membrane disruption is not evident. The apparent discrepancy between microscopy-based viability staining and XTT metabolic data, particularly in *C. parapsilosis*, underscores the complexity of biofilm responses to antimicrobial stress. While microscopy suggests a comparatively less affected architecture in *C. parapsilosis*, XTT assays reveal significant reduction in metabolic activity at 5 × MIC, indicating that cells within the biofilm remain metabolically impaired despite partial biofilm architecture preservation (Lohse et al. 2017).

Overall, these findings highlight that peptide-mediated antibiofilm activity involves multiple mechanisms, including disruption of biofilm structure, reduction of biomass, and impairment of cellular metabolic functions. The combined use of structural and functional assays is therefore essential to accurately assess peptide efficacy against mature biofilms, which exhibit intrinsic heterogeneity and adaptive responses to antimicrobial agents.

The in vitro toxicity assessments indicate a favorable safety profile for both lipidated peptides at concentrations relevant to antimicrobial activity. In murine 3T3 fibroblasts, cytotoxicity was confined to the highest tested doses, with Myr-T4 reducing cell viability at 64 and 32 µg/mL, whereas Cap-T4 was cytotoxic only at 64 µg/mL. Importantly, at concentrations ≤ 16 µg/mL for Myr-T4 and ≤ 32 µg/mL for Cap-T4, cell viability remained above the 70% ISO 10993–5 threshold, indicating acceptable biocompatibility (ISO 10993–5:^2009^). These data suggest that the capryl-modified peptide displays a slightly improved tolerability than the myristoylated analogue, likely reflecting differences in lipid chain length and membrane interaction dynamics.

These data, as well as the strong interaction established with the zwitterionic membrane used in the partition experiment, can be explained considering the length of the acyl chain used for the lipidation of the peptide sequence. In fact, an increase in hydrophobicity could lead to major toxicity in mammalian cells due to the loss of selectivity for microbial anionic membranes (Húmpola et al. 2017; Greber et al. 2017; Bellavita et al. 2023).

Our findings are in agreement with a study performed by Kamysz et al*.* showed that hemolysis of erythrocytes strongly depends on the number of carbon atoms in the fatty acid conjugated to the peptide sequence KRIVQRIKDFLR-NH₂ (Kamysz et al. 2020). Specifically, no hemolysis of human red blood cells was observed for sequences conjugated with acetic acid (C2) or butyric acid (C4). In contrast, hemolytic activity increased substantially for sequence functionalized with decanoic acid (C10) and myristic acid (C14). The authors further demonstrated that functionalization of KRIVQRIKDFLR-NH₂ with a longer acyl chain (C14) resulted in a much greater enhancement of hemolytic activity compared to antimicrobial activity. In fact, C14-KRIVQRIKDFLR-NH₂ showed no antimicrobial activity against any of the bacterial strains tested.

Finally, both peptides exhibited minimal toxicity against the *Galleria mellonella* model, with high larval survival across all tested concentrations and time points. Even at the highest dose, no significant reduction in survival was observed up to 72 h post-injection, supporting that the cytotoxic effects detected in vitro at elevated concentrations do not always translate into in vivo acute systemic toxicity. Taken together, these findings highlight a satisfactory therapeutic window for both LPs and support their further development as antimicrobial agents, identifying Cap-T4 as the more promising candidate in terms of safety profile.

Overall, the results reinforce lipidation effectiveness as a strategy for developing new antimicrobial drugs and emphasize the importance of balancing hydrophilicity and hydrophobicity in the design of synthetic peptides to ensure good biocompatibility when planning a new drug candidate.

## Conclusions

In conclusion, N-terminal lipidation markedly improved the antifungal activity of Trem-4, confirming this strategy as an effective approach to enhance the activity of short antimicrobial peptides against clinically relevant yeasts. Among the two analogues, Cap-T4 showed the most balanced profile, combining broad antifungal efficacy, better selectivity for anionic membrane models, and lower in vitro toxicity than Myr-T4. Myr-T4, although active against selected strains including *C. auris*, appeared more affected by medium-dependent activity and showed higher hemolytic and cytotoxic effects, likely due to its greater hydrophobicity and aggregation propensity.

 Both lipopeptides were also active against mature biofilms, where they reduced metabolic activity and disrupted biofilm architecture, although complete eradication was not achieved. Importantly, both compounds showed limited acute toxicity in *Galleria mellonella*, supporting a favorable in vivo tolerability. Overall, these findings identify Cap-T4 as the most promising candidate for further optimization and support the importance of finely tuning acyl-chain length to balance antifungal potency and host-cell selectivity.

## Supplementary Information

Below is the link to the electronic supplementary material.

ESM 1 (2.08 MB PDF)

## Data Availability

The original contributions presented in the study are included in the article/Supplementary material; further inquiries can be directed to the corresponding authors.
