# Supplementary material for: N-terminal lipidation enhances membrane interaction and antifungal activity of a Trematocine-derived decapeptide
Source: Appl Microbiol Biotechnol. 2026 Jun 29;110:277. doi: 10.1007/s00253-026-13929-4 (PMC13578187; doi:10.1007/s00253-026-13929-4)
Supplement: Supplementary file 1 [file 253_2026_13929_MOESM1_ESM.pdf]

Supplementary Information

N-terminal lipidation enhances membrane interaction and antifungal activity of a Trematocine-derived decapeptide

Damiano Squitieri [a*], Federica Massaro [b*], Melinda Mariotti [a*], Margherita Cacaci [a], Stefano Borocci[b], Fernando Porcelli[b], Francesco Buonocore [b§], Francesca Bugli [a§], Maurizio Sanguinetti [a,c]

[a] Department of Basic Biotechnological Sciences, Intensive and Perioperative Clinics, Catholic University of the Sacred Heart, L.go Francesco Vito 1, 00168 Rome (Italy)

[b] Department for Innovation in Biological, Agro-Food and Forest Systems (DIBAF), University of Tuscia, L.go dell'Università s.n.c., 01100 Viterbo (Italy)

[c] Department of Laboratory Sciences and Infectious Diseases, A. Gemelli University Hospital Foundation IRCCS, L.go Agostino Gemlli 8, 00168 Rome (Italy)

[§] Corresponding authors (fbuono@unitus.it; francesca.bugli@unicatt.it)

[*] Equally contributed

Supplementary MATERIALS AND METHODS

SM1. *In vitro* cytotoxic activity on *3t3* fibroblasts

Murine embryonic immortalized 3T3 fibroblasts were cultured at 37°C in a humidified environment (CO_2 5%) in DMEM containing L-glutamine, supplemented with 10% Fetal Bovine Serum (FBS) and 1% Penicillin-Streptomycin antibiotics. A total of 15,000 cells/well in medium were seeded into a 96-well plate until a sub-confluent monolayer was achieved. Cells were treated with different concentrations of the peptides to achieve the following final concentrations: 64, 32, 16, 8 µg/mL. Untreated cells were used as control and medium as blank. After 24h of incubation, cellular viability was evaluated by the XTT assay using the CyQUANT™ XTT Cell Viability Assay kit (Invitrogen, Thermo Fisher Scientific, Massachusetts, USA) according to the manufacturer's instructions. The OD, specific absorbance, and cellular viability were determined as previously described.

SM2. Certificate of analysis of Trem-4

Certificate of Analysis

For research use only!

Product:	Synthetic peptide
Lot No.:	P210323-01-05
Manufacturing date:	April 21st 2023
Peptide Sequence:	WKLLRGIVKV
Modifications:	None.
Format:	Lyophilized trifluoroacetate salt.
Theoretical M.W. of peptide:	1211.57
M.W. (M.W. + H^+) Measured by MS:	1213.01
M.W. Measured by MS:	1212.01
Match:	Approved.
Purity:	99.48%
Delivered quantity:	16.0 mg
HPLC results:	Enclosed.
Mass spec. results:	Enclosed.

CASLO ApS, c/o Technical University of Denmark, DTU-Science Park
Diplomvej 381, DK-2800, Kongens Lyngby, Denmark
Tel: +45- 70 23 28 60, Fax: +45- 70 23 29 90
E-mail: info@caslo.com Web page: www.caslo.com

HPLC Certificate

Lot No.	P210323-01-05
HPLC Column	(250×4.6mm I.D.) C18
Detection wavelength	220 nm
Gradient	20-50%B in 30 min
Buffer A	0.05%TFA +2%CH_3CN
Buffer B	0.05%TFA +90%CH_3CN

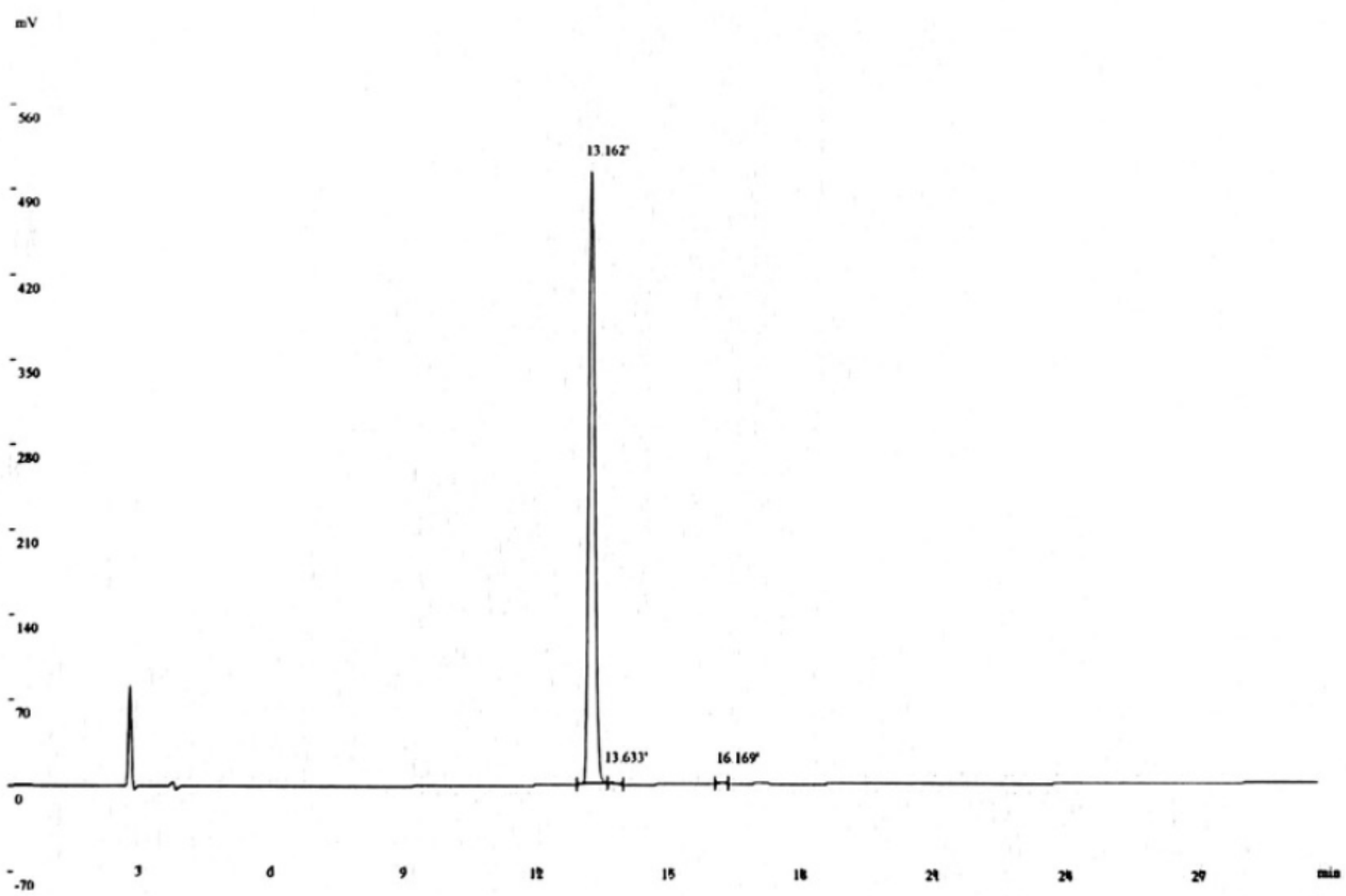

Peak Results

Rank	Time	Conc.	Area	Height
1	13.162	99.48	4791618	512522
2	13.633	0.3139	15119	1324
3	16.169	0.2043	9840	1169
Total		100	4816577	515015

Mass Spectrometry Certificate

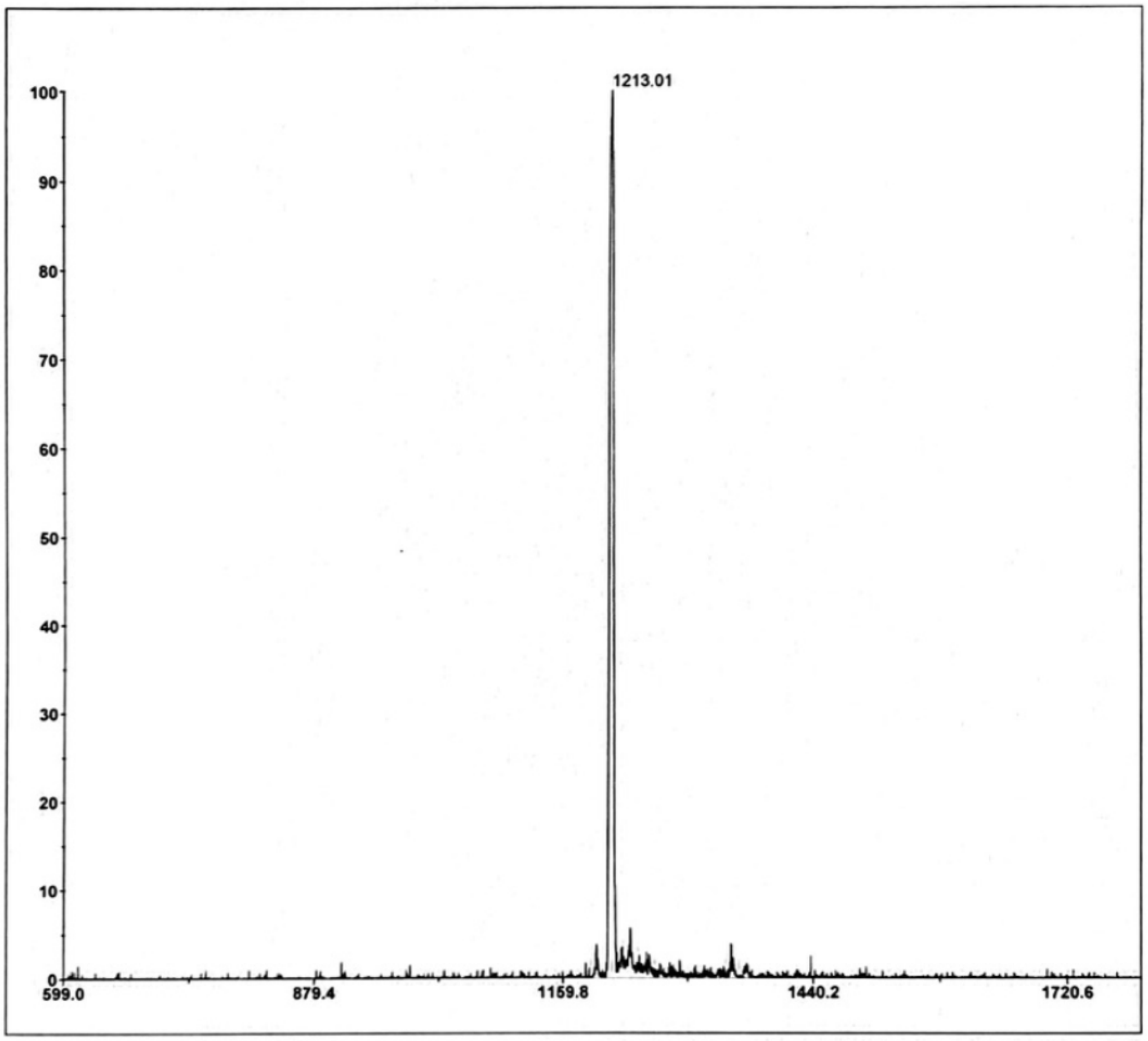

Lot No.: P210323-01-05
Method: MALDI-TOF
Main Peak: 1213.01
MW $[M+H^+]$: 1213.01
MW: 1212.01
Theoretical MW: 1211.57
Match: Approved
Z=1

SM3. Certificate of analysis of Cap-T4

Certificate of Analysis

For research use only!

Product:	Synthetic peptide
Lot No.:	P031225-01-04
Manufacturing date:	January 9th 2026
Peptide name:	Capr-Mut 4
Peptide sequence:	(Caprylic acid)-WKLLRGIVKV
Modifications:	Caprylic acid (C8) conjugated to the N-terminus.
Format:	Lyophilized trifluoroacetate salt.
Remarks:	None
Theoretical molecular weight (M.W.) of peptide:	1337.78
M.W. (M.W. + H^+) Measured by MS:	1338.54
M.W. Measured by MS:	1337.54
Match:	Approved.
Purity:	99.57%
Delivered quantity:	10.0 mg

CASLO ApS, c/o Technical University of Denmark, DTU-Science Park
Diplomvej 381, DK-2800, Kongens Lyngby, Denmark
Tel: +45- 70 23 28 60, E-mail: info@caslo.com
Web page: www.caslo.com

HPLC Certificate

Lot No.	P031225-01-04
HPLC Column	(250×4.6mm I.D.) C18
Detection wavelength	220 nm
Gradient	38-68%B in 30 min
Buffer A	0.05%TFA +2%CH_3CN
Buffer B	0.05%TFA +90%CH_3CN

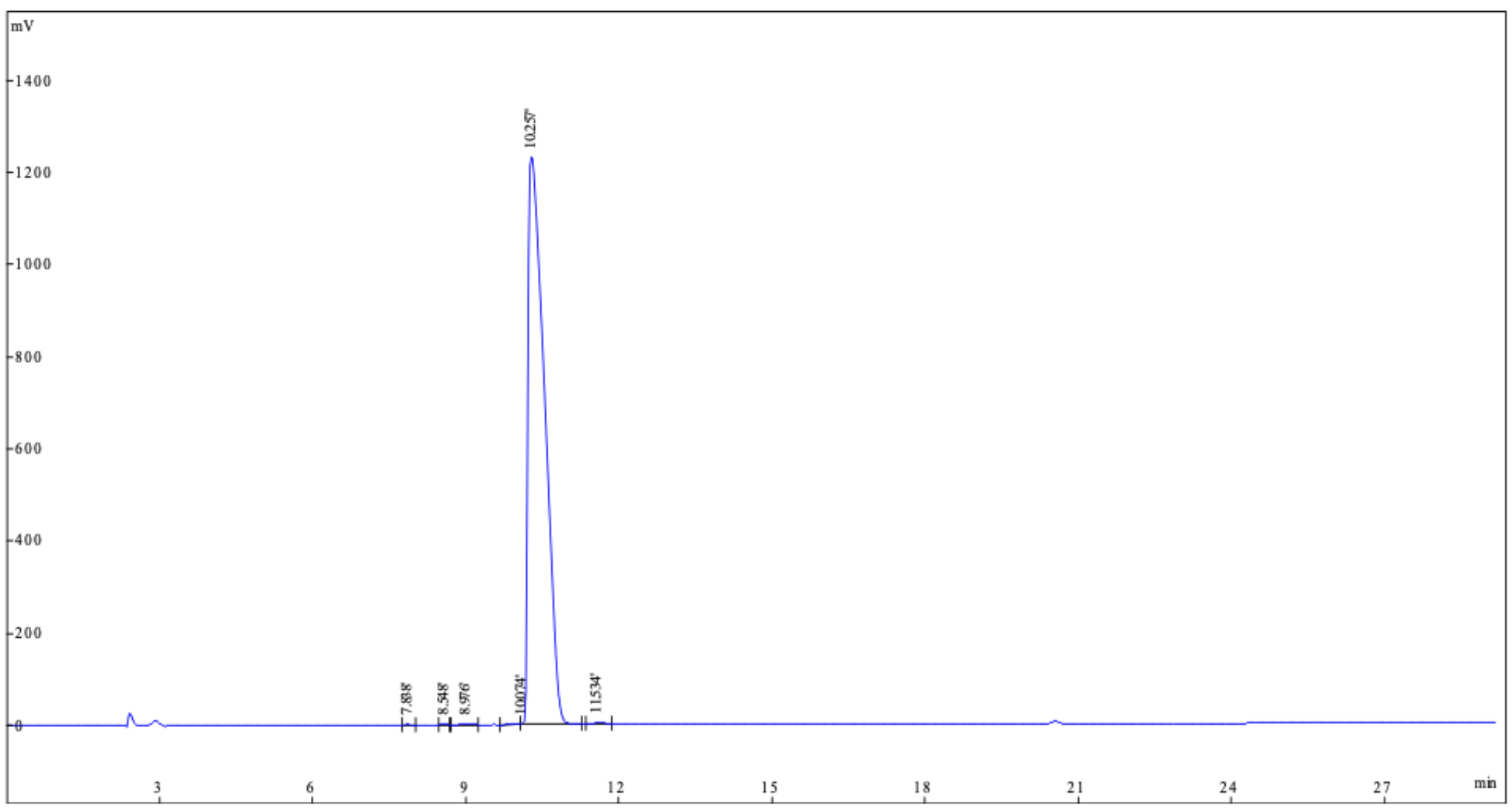

Peak Results

Rank	Time	Conc.	Area	Height
1	7.838	0.02056	5314	777
2	8.548	0.04458	11523	1697
3	8.976	0.09696	25064	1641
4	10.074	0.08919	23056	2650
5	10.257	99.57	25741869	1228713
6	11.534	0.1744	45080	2796
Total		100	25851906	1238274

Mass Spectrometry Certificate

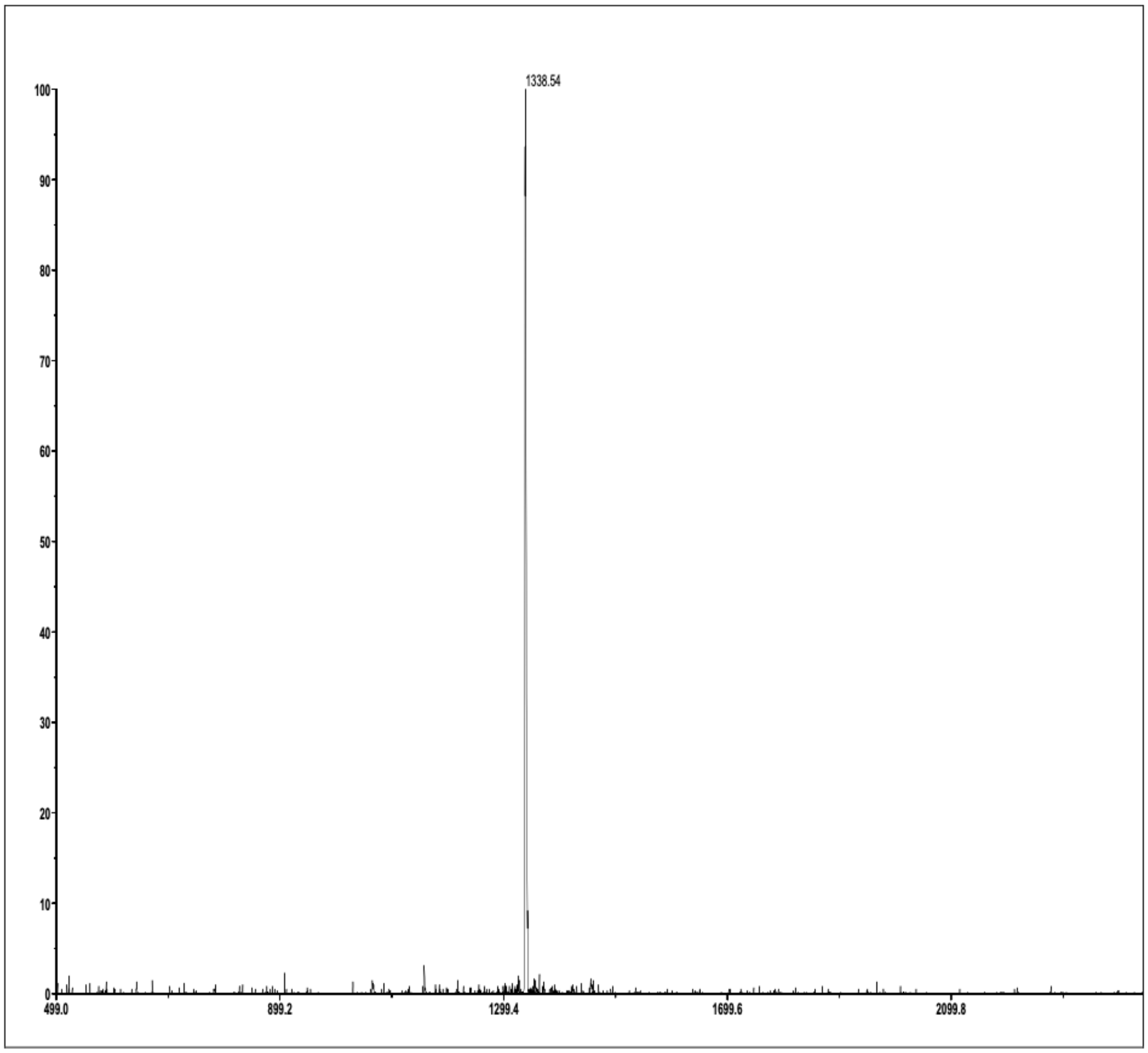

Lot No.: P031225-01-04
Method: MALDI-TOF
Main Peak: 1338.54
M.W. $[M+H^+]$: 1338.54
M.W.: 1337.54
Theoretical M.W.: 1337.78
Match: Approved
Z=1

SM4. Certificate of analysis of Myr-T4

Certificate of Analysis

For research use only!

Product:	Synthetic peptide
Lot No.:	P241023-01-02
Manufacturing date:	November 24th 2023
Peptide Sequence:	(Myristic acid)-WKLLRGIVKV
Modifications:	Myristoylated N-terminus.
Format:	Lyophilized trifluoroacetate salt.
Theoretical M.W. of peptide:	1421.95
M.W. (M.W. + H^+) Measured by MS:	1422.19
M.W. Measured by MS:	1421.19
Match:	Approved.
Purity:	96.75%
Delivered quantity:	10.0 mg
HPLC results:	Enclosed.
Mass spec. results:	Enclosed.

CASLO ApS, c/o Technical University of Denmark, DTU-Science Park
Diplomvej 381, DK-2800, Kongens Lyngby, Denmark
Tel: +45- 70 23 28 60, Fax: +45- 70 23 29 90
E-mail: info@caslo.com Web page: www.caslo.com

HPLC Certificate

Lot No.	P241023-01-02
HPLC Column	(250×4.6mm I.D.) C18
Detection wavelength	220 nm
Gradient	40-70%B in 30 min
Buffer A	0.05%TFA +2%CH_3CN
Buffer B	0.05%TFA +90%CH_3CN

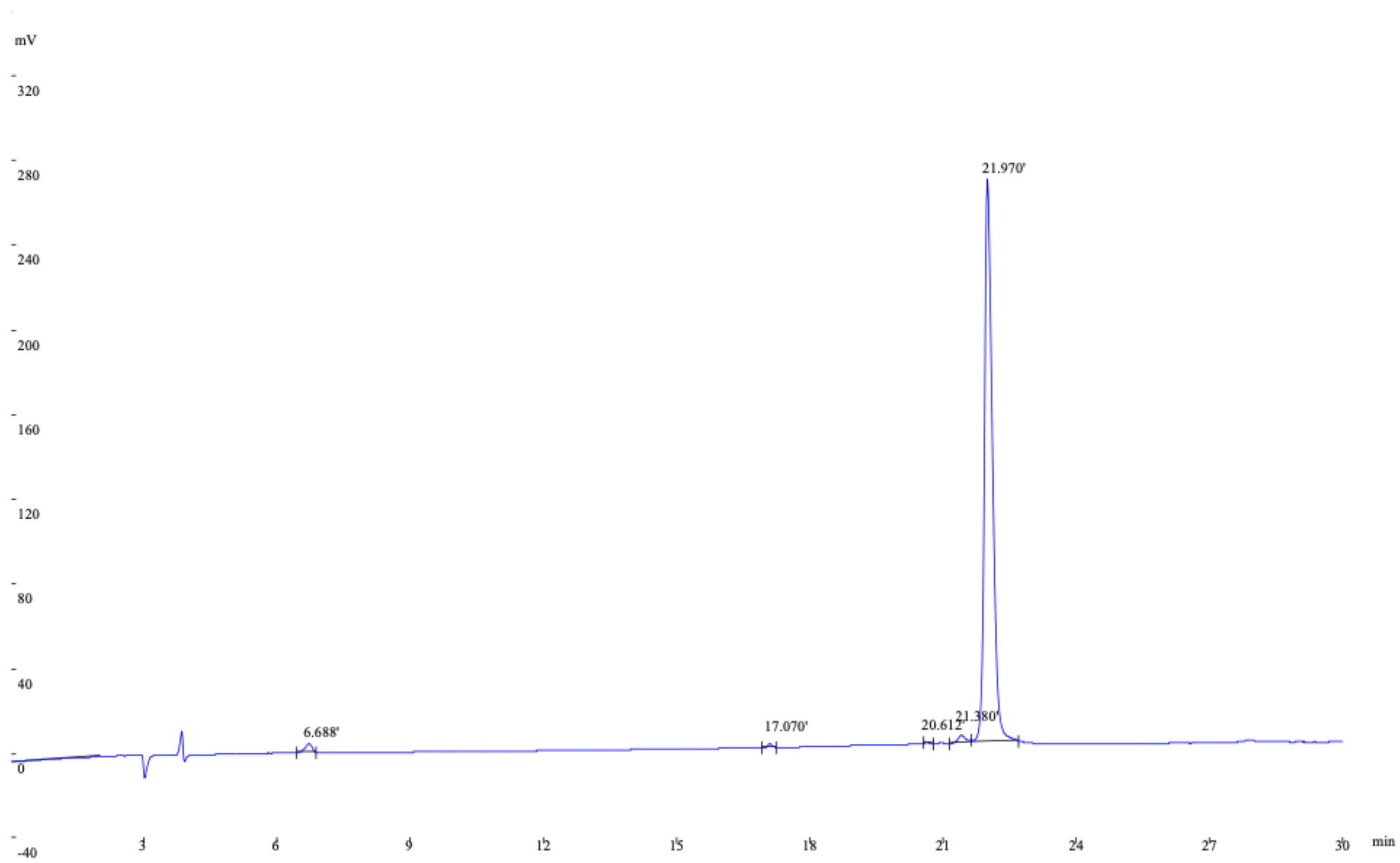

Peak Results

Rank	Time	Conc.	Area	Height
1	6.688	1.343	46171	4256
2	17.070	0.3414	11741	1667
3	20.612	0.05892	2026	527
4	21.380	1.5	51601	3734
5	21.970	96.75	3327460	266515
Total		100	3438999	276699

Mass Spectrometry Certificate

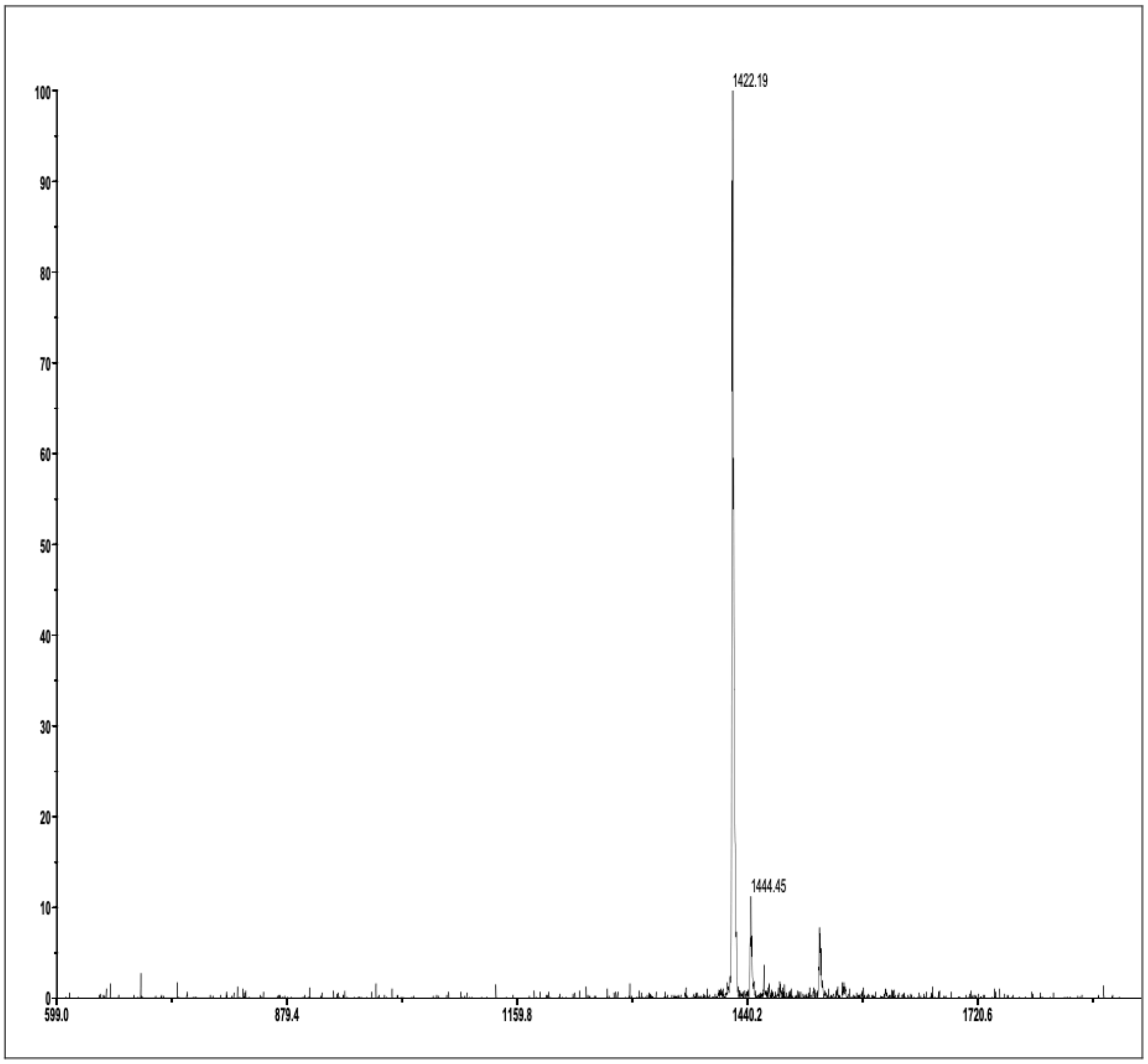

Lot No.: P241023-01-02
Method: MALDI-TOF
Main Peak: 1422.19
MW [M+H$^+$]: 1422.19
MW: 1421.19
Theoretical MW: 1421.95
Match: Approved
Z=1

Supplementary RESULTS

Supplementary Table S1 Antifungal susceptibility profiles of clinical isolates

***Candida albicans* (n = 11)**

Drug	MIC median (range, µg/mL)	%R (CLSI)
Anidulafungin	0.016 (≤0.016–0.03)	0
Micafungin	0.016 (0.008–0.016)	0
Caspofungin	0.03 (0.016–0.06)	0
Isavuconazole	0.008 (≤0.008–1)	0
Posaconazole	0.03 (0.016–0.5)	0
Voriconazole	0.016 (≤0.008–2)	27.3
Itraconazole	0.03 (0.016–0.5)	27.3
Fluconazole	0.25 (0.25–64)	27.3
Amphotericin B	0.25 (≤0.125–0.5)	0

***Candida glabrata* (n = 11)**

Drug	MIC median (range, µg/mL)	%R (CLSI)
Anidulafungin	0.03 (0.016–0.06)	0
Micafungin	0.016 (0.016–0.016)	0
Caspofungin	0.06 (0.03–0.25)	0
Isavuconazole	0.5 (0.25–8)	0
Posaconazole	2 (1–>8)	0
Voriconazole	0.5 (0.25–>8)	0
Itraconazole	1 (0.5–>16)	0
Fluconazole	16 (8–256)	36.4
Amphotericin B	0.5 (≤0.125–1)	0

***Candida tropicalis* (n = 10)**

Drug	MIC median (range, µg/mL)	%R (CLSI)
Anidulafungin	0.016 (0.016–0.03)	0
Micafungin	0.03 (0.03–0.06)	0
Caspofungin	0.03 (0.016–0.06)	0
Isavuconazole	0.125 (0.06–2)	0
Posaconazole	0.125 (0.06–0.5)	0
Voriconazole	0.125 (0.06–4)	27.3
Itraconazole	0.25 (0.125–0.5)	27.3
Fluconazole	2 (1–64)	27.3
Amphotericin B	0.25 (0.125–0.5)	0

***Candida parapsilosis* (n = 11)**

Drug	MIC median (range, µg/mL)	%R (CLSI)
Anidulafungin	0.5 (0.25–2)	0
Micafungin	0.5 (0.25–2)	0
Caspofungin	0.25 (0.06–0.5)	0
Isavuconazole	0.016 (0.008–0.125)	0
Posaconazole	0.03 (0.016–0.06)	0
Voriconazole	0.06 (0.008–2)	36.4
Itraconazole	0.06 (0.016–0.125)	45.5
Fluconazole	1 (0.25–>256)	36.4
Amphotericin B	0.25 (≤0.125–1)	0

***Candidozyma auris* (n = 10)**

Drug	MIC median (range, µg/mL)	%R (CLSI)
Anidulafungin	0.125 (0.06–4)	10
Micafungin	0.125 (0.06–>8)	10
Caspofungin	0.25 (0.125–4)	10
Isavuconazole	2 (0.125–2)	0
Posaconazole	0.5 (0.06–1)	0
Voriconazole	4 (0.5–8)	0
Itraconazole	0.5 (0.125–1)	0
Fluconazole	>256 (64–>256)	100
Amphotericin B	0.5 (≤0.125–2)	10

***Cryptococcus neoformans* (n = 6)**

Drug	MIC median (range, µg/mL)	%R (CLSI)
Isavuconazole	0.25 (0.06–0.5)	NA
Posaconazole	0.5 (0.25–0.5)	NA
Voriconazole	0.5 (0.125–1)	NA
Itraconazole	0.25 (0.125–0.5)	NA
Fluconazole	32 (8–32)	NA
Amphotericin B	0.25 (0.125–0.5)	NA

***Candidozyma vulturna* (n = 1)**

Drug	MIC median (range, µg/mL)	%R (CLSI)
Anidulafungin	0.125 (0.125–0.125)	NA
Micafungin	0.125 (0.125–0.125)	NA
Caspofungin	0.125 (0.125–0.125)	NA
Isavuconazole	0.06 (0.06–0.06)	NA
Posaconazole	0.125 (0.125–0.125)	NA
Voriconazole	0.125 (0.125–0.125)	NA
Itraconazole	0.125 (0.125–0.125)	NA
Fluconazole	8 (8–8)	NA
Amphotericin B	2 (2–2)	NA

MIC values were determined according to CLSI guidelines. percent resistance (%R) was calculated based on CLSI clinical breakpoints where available. For *Cryptococcus neoformans*, echinocandins are considered intrinsically inactive, and no breakpoints are defined (NA). ND, not determined; MIC, minimum inhibitory concentration; CLSI, Clinical & Laboratory Standards Institute.

Supplementary Table S2 MIC and MFC values of Trem-4 lipopetide against representative fungal clinical isolates.

Yeast strain	MIC (µg/mL)	MFC (µg/mL)
C. albicans R	64 ± ND	64 ± ND
C. albicans S	64 ± ND	64 ± ND
C. glabrata R	64 ± ND	64 ± ND
C. glabrata S	64 ± ND	64 ± ND
C. tropicalis S	64 ± ND	64 ± ND
C. tropicalis R	64 ± ND	64 ± ND
C. parapsilosis R	64 ± ND	64 ± ND
C. parapsilosis S	64 ± ND	64 ± ND

C. auris R	64 ± ND	64 ± ND
C. auris S	64 ± ND	64 ± ND
C. neoformans R	64 ± ND	64 ± ND
C. neoformans S	64 ± ND	64 ± ND
C. vulturna	64 ± ND	64 ± ND

ND, not determined; MIC, minimum inhibitory concentration; MFC, minimum fungicidal concentration; R, resistant; S, susceptible as defined in materials and methods section.

Fig. S1

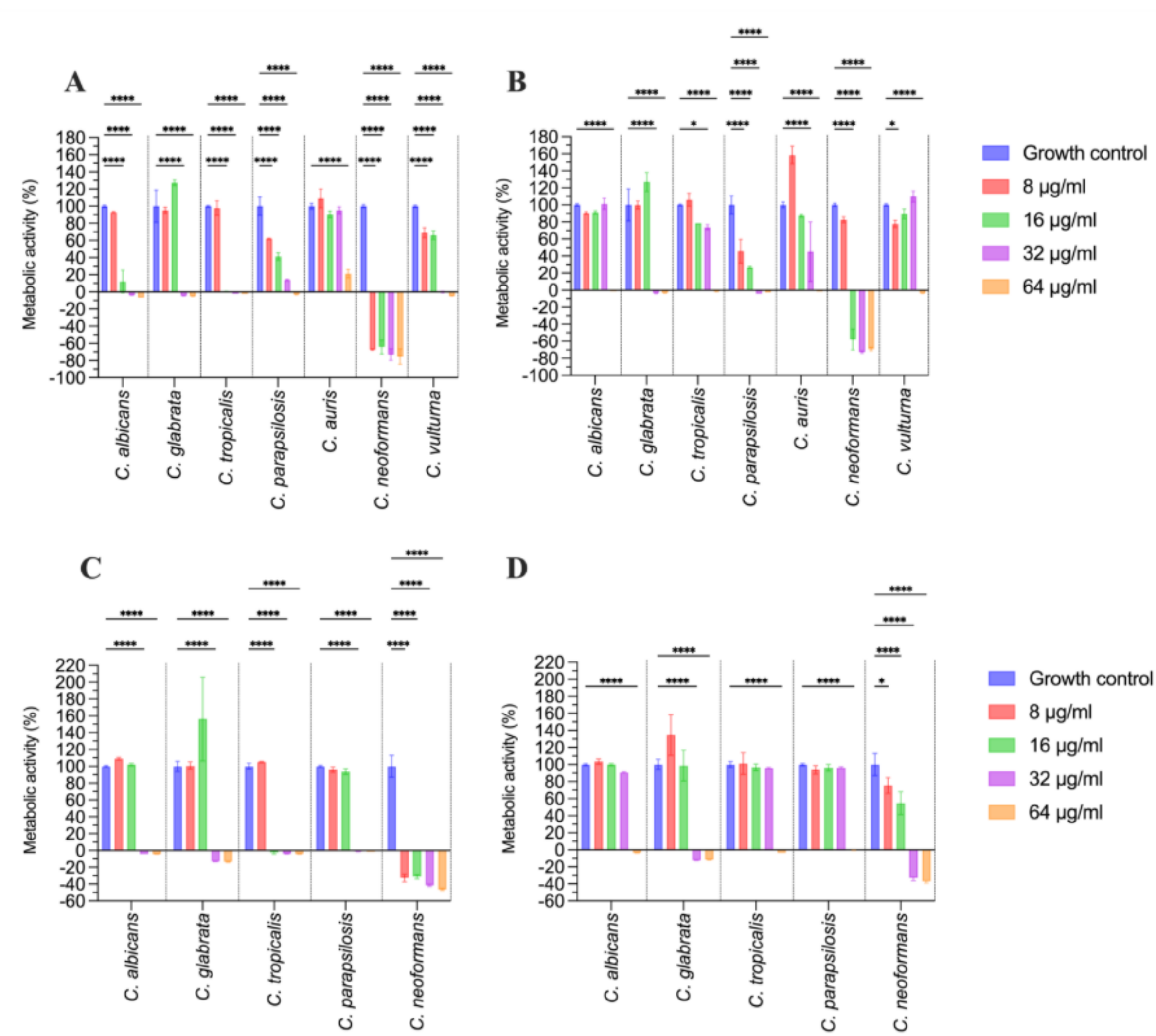

Metabolic activity of planktonic *Candida* spp. and *C. neoformans* cells assessed by XTT assay after 24 hours of broth microdilution (BMD). Planktonic cells were exposed to the tested peptides during standard BMD assays. Panels A and B report results obtained on selected antifungal-resistant strains, while panels C and D refer to antifungal-susceptible

strains. Panels A and C show treatment with Cap-T4, whereas panels B and D show treatment with Myr-T4. Metabolic activity was quantified by XTT reduction and expressed as percentage relative to untreated growth controls. Data are reported as mean ± SD. Statistical significance was calculated relative to the growth control condition; *$p < 0.05$ and ≥ 0.01, **$p < 0.01$ and ≥ 0.001, ***$p < 0.001$ and ≥ 0.0001, ****$p < 0.0001$.

Fig. S2

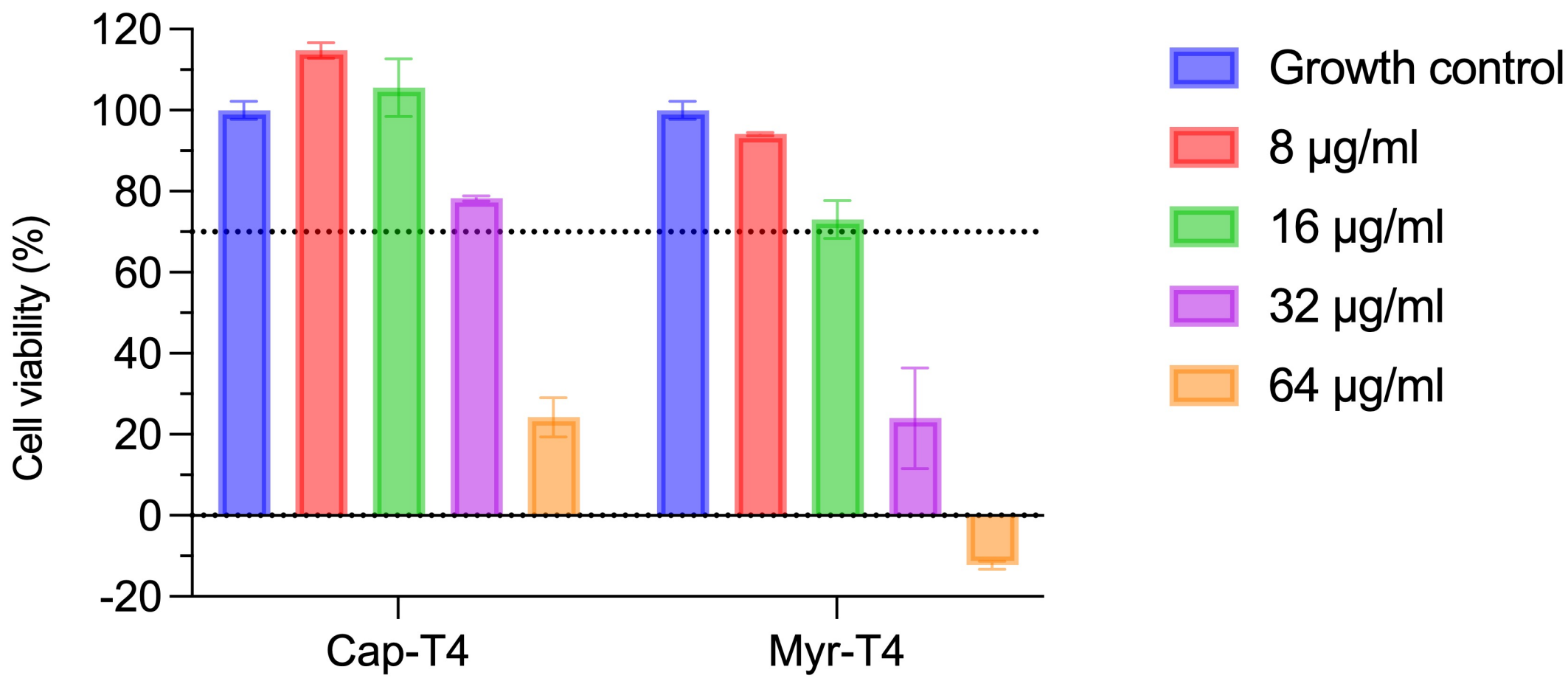

In vitro cytotoxicity on murine embryonic immortalized 3T3 fibroblasts after 24 h of treatment, evaluated by XTT assay. Data are normalized to untreated controls. The horizontal dotted line indicates the 70% cell viability threshold according to ISO 10993-5:2009.
